# Blueprint for clinical N-of-1 strategies with off-label precision treatments in monogenic epilepsies

**DOI:** 10.1186/s13023-025-03750-z

**Published:** 2025-06-16

**Authors:** Victoria M. Defelippe, Eva H. Brilstra, Willem M. Otte, Ghislaine J. M. W. van Thiel, Helen J. Cross, Finbar O’Callaghan, Valentina De Giorgis, Emilio Perucca, Kees P. J. Braun, Floor E. Jansen

**Affiliations:** 1 Department of Child Neurology, Brain Center University Medical Center Utrecht, Universiteitsweg 100, 3584 CG Utrecht, The Netherlands; 2 Department of Genetics, Brain Center University Medical Center Utrecht, Universiteitsweg 100, 3584 CG Utrecht, The Netherlands; 3 Department of Medical Humanities, Julius Center for Health Sciences and Primary Care, University Medical Center Utrecht, Universiteitsweg 100, 3584 CG Utrecht, The Netherlands; 4 Developmental Neurosciences, University College London (UCL) Great Ormond Street NIHR BRC, Institute of Child Health, 30 Guilford Street, London, WC1 N 1EH UK; 5 Fondazione Mondino National Institute of Neurology/University of Pavia, Via Mondino 2, 27100 Pavia, Italy; 6 Department of Medicine, University of Melbourne (Austin Health), Heidelberg, VIC 3084 Australia; 7 Australia and Department of Neuroscience, Monash University, Melbourne, VIC Australia

**Keywords:** N-of-1 trial, Single case-studies, Precision medicine, Quality improvement of care, Epilepsy

## Abstract

Precision treatments for monogenic epilepsies, i.e. treatments that can at least partially reverse the biochemical consequences of a pathogenic gene variant, have been gradually emerging over the years. To date, however, information on the efficacy of these treatments is mostly based on case-reports and retrospective studies. As a result, utilisation of precision treatments often lack consistency and a pre-defined outcome monitoring plan. N-of-1 strategies in clinical care are pre-defined, individually tailored, repeated challenge-withdrawal therapeutic trials designed to assess the value of a treatment of interest for an individual. Despite their potential to improve clinical decision-making, N-of-1 strategies have been hampered by limited guidance on their implementation and lack of consensus on oversight procedures. To improve treatment selection for rare monogenic epilepsies, the PINPOINT initiative (Precision Treatments In MoNogenic EPilepsies: Observational Registry And N-of-1 Trial Recommendations) was set up as a collaborative effort within the European Reference Network for Rare and Complex Epilepsies. PINPOINT aims to develop recommendations for the design of N-of-1 strategies with off-label precision treatments for monogenic epilepsies. Using available N-of-1 trial manuals, different components of N-of-1 design were tailored to the context of epilepsy and oversight procedures were outlined. These efforts resulted in this guidance document—or blueprint for N-of-1 strategies for monogenic epilepsies in clinical care. This blueprint defines the characteristics of treatments and patients that would be suitable for N-of-1 strategies. Key principles for outcome measure selection, period duration and statistical analysis are defined. Consideration is given to interim assessment rules, which establish whether proceeding onto an additional treatment cycle is likely to provide significant advantages. Procedures for ethical oversight are proposed. This blueprint for N-of-1 strategies can be used as a basis for master protocols to optimise individualised clinical care in a standardised and consistent manner. We are confident that this document will provide physicians with the building blocks needed to elevate precision treatments for rare monogenic epilepsies out of their current landscape of inadequate evidence.

**Supplementary Information:**

The online version contains supplementary material available at 10.1186/s13023-025-03750-z.

## Background

Approximately 25–40% of epilepsies with onset in infancy and early childhood are caused by a single gene variant [1–4]​. Understanding the molecular mechanisms underlying monogenic epilepsies has accelerated the discovery of precision treatments [5–9]​​, i.e. targeted treatments which can at least partially correct the dysfunction caused by the specific gene defect [6, 10]. These treatments may include already marketed antiseizure medications (ASMs) as well as re-purposed drugs that have been licensed for other indications. Unfortunately, efforts to conduct randomised controlled trials (RCTs) to inform treatment recommendations for most of the monogenic epilepsies are hampered by their inherent low prevalence and interpatient heterogeneity. Evidence of the effectiveness of precision treatments for the majority of monogenic epilepsies is based mainly on case-reports or retrospective studies [1, 7, 10]. As a result, while various precision off-label treatments have emerged as potential therapeutic alternatives for the treatment of monogenic epilepsies, evidence for their effectiveness has not been clearly established, which in turn hinders the implementation of these treatments in clinical care.

Currently, off-label treatments are prescribed through a pragmatic, but highly subjective, ‘trial and error’ approach. Decisions to continue or stop the treatment after a certain time interval are based on the shared evaluation of benefits and adverse effects as reported by patients or caregivers. In short, these therapeutic trials lack consistency in implementation and do not have a predefined monitoring plan nor an objective assessment of clinical outcomes. Inadequate assessment of treatment efficacy may prolong the therapeutic odyssey for patients. A more structured and rationally designed implementation of off-label precision treatments could improve patient outcomes by ensuring optimal use of these treatments and a better assessment of their actual effectiveness in routine clinical care.

**Fig. 1:**
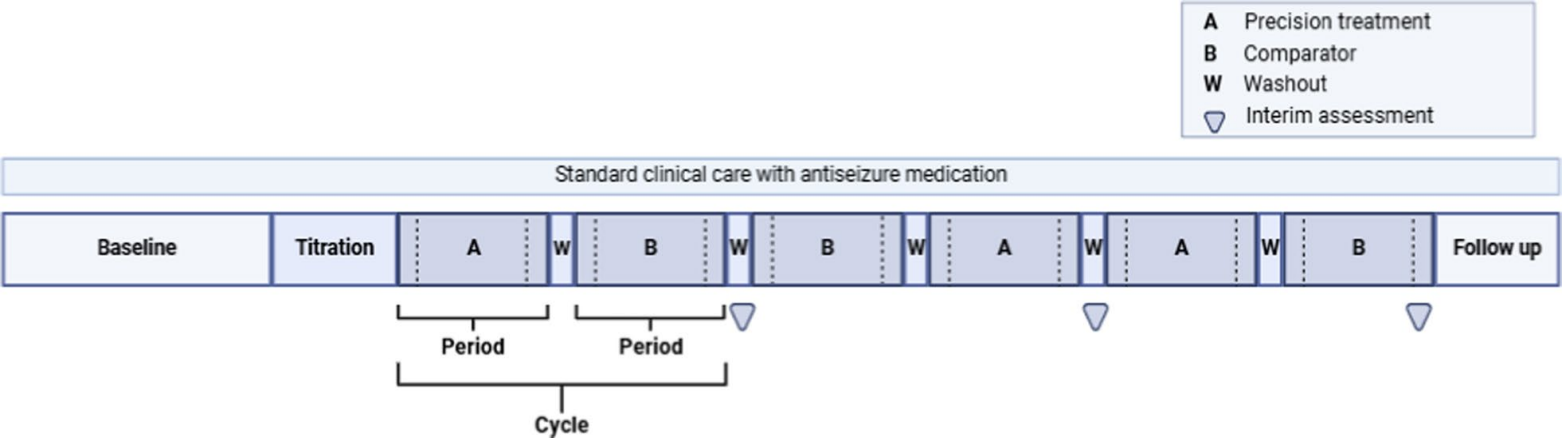
Schematic representation of N-of-1 strategy in rare monogenic epilepsies. An N-of-1 strategy involves one or more cycles composed of paired periods, e.g. (A) active treatment of interest, in this case a precision treatment and (B) a comparator which could be placebo, another active treatment or standard clinical care. Each cycle also includes a (W) washout interval between interventions. The dotted line at the end and start of each period represent titration and tapering phases. At the end of each cycle an interim assessment takes place to decide whether an additional cycle would be required. Throughout the N-of-1 therapy plan the underlying treatment with antiseizure medication remains unchanged. Created in https://BioRender.com

An N-of-1 strategy (also referred to as an N-of-1 trial), is a predefined treatment plan with repeated measurements during alternating periods of an active intervention and a comparator. Its purpose is to assess the efficacy and tolerability of a treatment for an individual (Fig. 1; Box 1) [11–13]. N-of-1 strategies have recently been rediscovered as an alternative to RCTs in small patient populations or in populations with heterogeneity of treatment effects [14–16]. Originally, N-of-1 trials were developed as a method to assess the value of a treatment for an individual in cases of clinical equipoise [11, 15]. In clinical practice, the N-of-1 approach can help to assess individual responsiveness to an intervention to prevent either unnecessary continuation of an ineffective treatment or premature discontinuation of an effective intervention.

**Box 1:** N-of-1 strategy terminology

Term	Definition
N-of-1 strategy in clinical care	An N-of-1 strategy designed to improve clinical care of an individual patient. The strategy involves assessment of treatment safety and efficacy using alternating periods with the treatment of interest and a comparator (control). A predefined treatment and monitoring plan and outcome measurements are applied. Also referred to here as: N-of-1 treatment (or therapy) plan (protocol)
Research N-of-1 strategy	An N-of-1 strategy conducted for the purpose of scientific research
Period	Predefined interval during which an intervention or other components of the study design are applied. Examples include the active treatment period and the placebo period. The duration of each period may vary according to the characteristics of the patient and the treatment to be assessed. Each period may be divided into phases, for example a treatment period may include a titration, a maintenance and a tapering phase. Periods may be separated by a washout interval
Cycle	A repeated unit including two or more fixed periods. The sequence of periods may or may not be in random order
Comparator or control	Another treatment (e.g. placebo or another active treatment) or an interval without treatment which serves as a reference against which the effect of the treatment of interest can be assessed (compared)
Crossover	Alternating allocation to different treatment(s) and comparator sequentially in one individual
Carryover	When treatment effects linger beyond the crossover
Titration phase	Time interval during which the dose is adjusted upwards gradually (titration)
Tapering phase	Time interval during which the dose is adjusted downwards gradually (down-titration)
Washout interval	Interval in which no treatment of interest or comparator are administered, to permit cessation of the effect of the previous treatment prior to initiation of another treatment period
Counterbalanced randomization	Randomly generated sequence of periods with treatment and comparator (or control situation) designed to balance the assignment to both within and across cycles
Interim assessment	Predefined assessments at specified times to determine whether to proceed or terminate the N-of-1 strategy
Stopping rules	Predefined rules stating conditions under which the N-of-1 strategy should cease because of lack of effectiveness, adverse effects, overwhelming evidence of superiority of one of the treatments, or other conditions which would make ethically unjustifiable to continue with the strategy

N-of-1 strategies are suitable for stable or slowly progressive chronic conditions with recurrent symptomatic events such as epilepsy, and with treatments with rapid and reversible effects [17, 18]. Recent reports highlight the challenges in distinguishing treatment-related clinical improvement from natural fluctuations in symptom severity in epilepsy trials and in clinical care [19–21]. The use of N-of-1 strategies in which the treatment of interest and placebo or another comparator alternate over time may counterbalance the influence of random variation in symptoms when assessing treatment effectiveness. For individuals with very frequent seizures, in particular, N-of-1 strategies allow to ascertain treatment effectiveness robustly over a relatively short period. Despite their promise in the context of seizure disorders, few N-of-1 trials in epilepsy have been conducted to date [18, 22], probably due to the presence of practical and regulatory barriers [13]. Here, we aim to provide a step-by-step guide on how to apply N-of-1 strategies in clinical practice to facilitate a more systematic assessment of the efficacy and safety of precision therapies for individuals with monogenic epilepsies, and we propose a framework for institutional oversight procedures. We provide a clinical case as a stepwise example of how to apply these recommendations (Box 2–6 throughout text). 

## Objectives

Building on the equipoise which results from the uncertainty about the actual value of most precision treatments for monogenic epilepsies, this blueprint aims to facilitate the transition from the classic ‘trial and error’ treatment approach to responsible clinical N-of-1 strategies. By providing physicians with the knowledge and tools needed to conduct N-of-1 strategies, this blueprint can serve as a basis for master protocols to guide individualised treatment design as well as corresponding oversight procedures.

Specifically, this blueprint will:

1. Outline the characteristics of treatments and patients for which and to whom clinical N-of-1 strategies may be applied;
2. Outline procedures and principles to assess the potential risks and benefits of clinical N-of-1 strategies;
3. Provide a toolbox of methodologically sound approaches that can be readily applied by the clinician.

## Methods

In response to the need to improve the quality of current trials with precision treatments [1, 7, 23], a collaborative effort within the European Reference Network for Rare and Complex Epilepsies (ERN EpiCARE) established the PINPOINT initiative (Precision Treatments In MoNogenic EPilepsies: Observational Registry And N-of-1 Trial Recommendations). The aim of PINPOINT is to develop N-of-1 strategy recommendations for off-label and new precision treatments for monogenic epilepsies. Using our preliminary analysis of the registry on clinical experiences with off-label precision treatments for monogenic epilepsies within the EpiCARE centres we identified rrelevant components for the N-of-1 therapy plan, such as (non-)seizure outcomes, and shortcomings of the ‘trial and error’ treatment approach which we address in the blueprint. Our recent systematic review of N-of-1 strategies in epilepsy [18] highlighted key methodological aspects which were used as a basis for this blueprint.

**Fig. 2:**
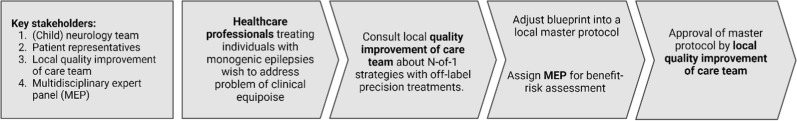
Schematic representation of proposed workflow of the blueprint as a master protocol for a quality improvement of care project. A team of healthcare professionals treating individuals with monogenic epilepsies who encounter situations of clinical equipoise can coordinate efforts with patient representatives to address this problem. The team of healthcare professionals can consult the quality improvement of care team locally about implementing clinical N-of-1 strategies with off-label precision treatments. According to the local advice the blueprint could be adjusted into a master protocol. A multidisciplinary expert panel (MEP) should be assigned locally, or if lacking local expertise, internationally. The master protocol should be submitted for approval by the local quality improvement of care team. This proposed workflow may vary according to local regulations

In view of the unmet needs in the treatments of monogenic epilepsies [5, 23, 24] and the lack of consensus regarding ethical oversight of N-of-1 strategies [13, 25–28], the authors consulted with the Dutch Central Committee on Research Involving Human Subjects (CCMO) in several meetings spanning from April 2021 up to December 2023. This resulted in a proposed ethical framework of oversight procedures for N-of-1 strategies for clinical care and research [29]. Superimposing mandatory institutional review board (IRB) reviews and other research regulations onto N-of-1 strategies in clinical care management leads to disproportionate financial, logistical and administrative hurdles (summarised in Appendix 1) despite these regulations not having been designed for care practices [30]. This blueprint refers solely to ‘N-of-1 strategies’ aimed at improving clinical decision-making. Therefore, unless stated otherwise, the term ‘N-of-1 strategies’ in the sections below refers to use of these strategies in clinical care and not in research. While our primary focus was not on research, existing guidelines/recommendations for N-of-1 trials, namely SPIRIT (the Standard Protocol Items: Recommendations for Interventional Trials), SPENT (the SPIRIT extension for N-of-1 trials checklist 2019), CONSORT (consolidated reporting items for trials) and CENT (the CONSORT extension for N-of-1 trials) [13, 31, 32] were used by the PINPOINT team as guiding principles for developing this blueprint, which has been endorsed as part of a master protocol for quality improvement of care at University Medical Centre (UMC) Utrecht. Figure 2 depicts a schematic representation of the workflow proposed here in which a healthcare team first consults their quality improvement of care team for local approval to conduct clinical N-of-1 strategies, provided that conditions outlined in this protocol have been met. Appendix 2 includes a feasibility and benefit-risk assessment form as the first steps to check whether an N-of-1 strategy would be plausible and justified as a quality improvement of care initiative (Appendix 2).

**Fig. 3:**
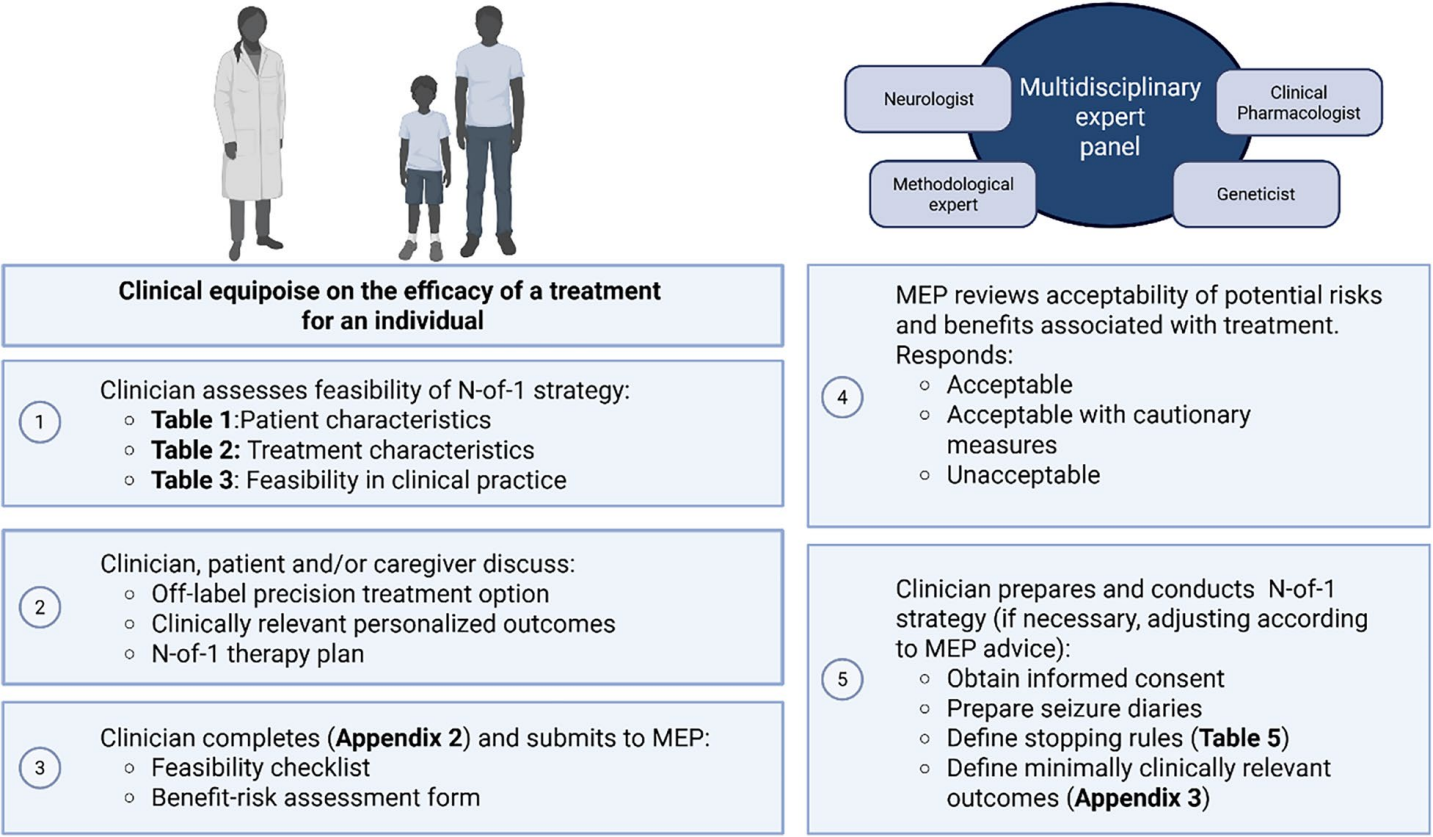
Schematic representation of steps to be followed by clinicians to conduct N-of-1 strategy in individual cases of equipoise. After obtaining approval to conduct clinical N-of-1 strategies according to the guiding principles outlined in this blueprint (and local recommendations), a clinician who encounters a situation in which the value of a precision treatment for an individual is unknown/doubtful and wishes to address to evaluate this treatment with an N-of-1 strategy can follow the steps listed here. First, feasibility of an N-of-1 strategy according to patient characteristics, suitability of the treatment and practical aspects can be assessed as outlined in Tables 1, 2 and 3. Next, a preliminary N-of-1 plan can be discussed with patient and/or caregiver. Third, the clinician completes the documents required to formally assess feasibility and potential benefits and risks associated with the treatment and submits these to the MEP. The MEP responds with their advice which can involve additional cautionary measures or deem the proposed treatment to have unacceptable risks. Finally, according to the advice from MEP, the clinician can finalise the N-of-1 therapy plan defining individualised outcomes with the patient and/or caregiver, obtain informed consent and conduct the N-of-1 treatment plan

The toolbox to aid physicians in developing methodologically sound N-of-1 strategies (Appendix 3) is composed of three parts: (A) defining treatment period duration, (B) selecting outcomes of interest and (C) randomisation, blinding and statistical analyses. Part A is based on N-of-1 trial manuals that define treatment duration for frequency-based outcomes [13, 17, 18] and literature on reliable reporting of seizures [13], taking into account the patient characteristics, pharmacokinetic factors and other relevant variables (need for titration, time required to achieve measurable clinical effects) [33, 34]. Part B was developed by reviewing literature on outcomes of interest for monogenic epilepsies and on assessment tools relevant for N-of-1 strategies. Part C provides options for statistical analyses in accordance with N-of-1 trial manuals [13]. Figure 3 shows a schematic representation of the steps to be followed by the clinician (after approval of a master protocol) to conduct responsible clinical N-of-1 strategies to assess the value of a precision treatment for an individual with monogenic epilepsy.

## Results

### Target population

Clinical equipoise is defined as a situation when “an honest, professional disagreement among expert clinicians about the preferred treatment” exists [35]. Clinical equipoise is ubiquitous in the care for patients with rare complex epilepsies. As a result, for the majority of rare monogenic epilepsies, there are no treatment guidelines available (or the patient does not fit existing guidelines), and there is no scientific evidence to determine the effectiveness of a treatment for a patient with specific characteristics. Box 2 describes a clinical scenario in which true clinical equipoise exists and an N-of-1 therapy plan would be considered. N-of-1 strategies can be applied to patients and treatments with predefined characteristics (Tables 1 and 2), provided that clinical equipoise conditions are met. Patient characteristics and criteria to ensure safe, responsible and methodologically sound N-of-1 strategies are defined below (Table 1). For patients with monogenic epilepsies, we consider only those with (likely) pathogenic genetic variants, as defined by the American College of Medical Genetics (ACMG) guidelines, could potentially benefit from an N-of-1 therapy plan with a potential precision therapy. Patients with recent treatment changes must have returned to a stable situation to allow a reliable assessment of treatment effectiveness in the context of an N-of-1 treatment plan. Additionally, N-of-1 strategies are only suitable for patients and for treatments that permit assessment of effectiveness over relatively short periods of time.

**Box 2:**
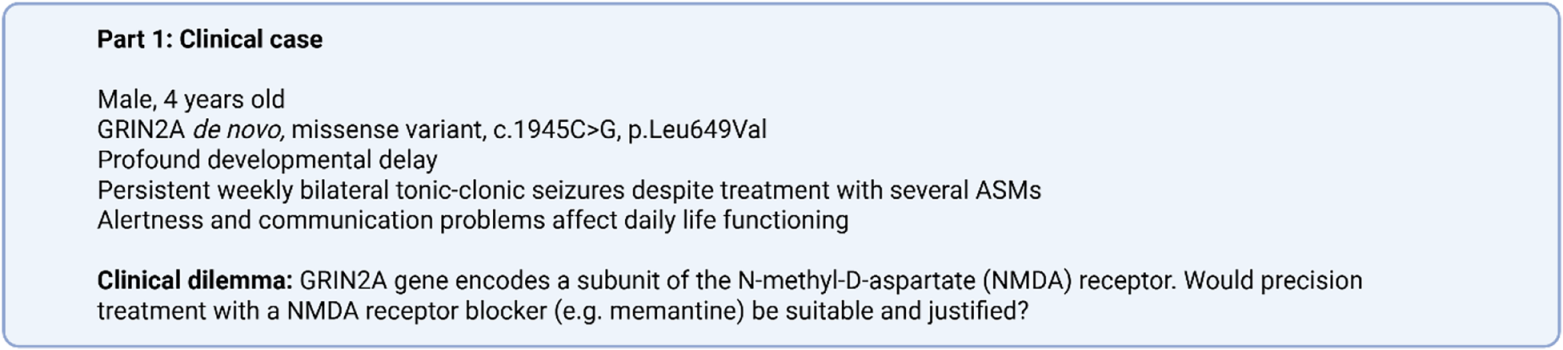
Clinical case of refractory epilepsy and clinical equipoise on potential precision treatment option

**Table 1:** Characteristics of patients who may benefit from clinical N-of-1 strategies

Patient specific characteristics	
*General characteristics:* •Infants > 3 months, children and adults with clinical diagnosis of epilepsy made by a neurologist or paediatric neurologist according to the definition of the International League Against Epilepsy 2017 AND •Monogenic epilepsy, i.e. epilepsy caused by a single-gene pathogenic variant classified as Class IV or V according to the American College of Medical Genetics (ACMG) guidelines AND •Patients for whom the licensed treatments are known to be ineffective or not indicated, or have been adequately tried without achieving adequate control of seizures or other clinically relevant comorbid symptoms related to the gene defect AND •An off-label precision treatment is available which is likely to correct the consequences of the patient’s gene defect	

Criteria for suitable situations for N-of-1 strategies	Criteria to avoid N-of-1 strategies
•Patient shows consistent therapy adherence •Patient and/or caregivers are interested in the N-of-1 approach for care and provide informed consent to participate in the N-of-1 strategy •Recurrent seizures with at least two or more measurable seizures per week (not on the same day) OR other non-seizure symptoms measurable over short periods (e.g. behaviour problems, motor function, spike-wave index on EEG) •Consistently present parent or proxy capable to define and report personalised treatment outcomes •If ketogenic diet or vagus nerve stimulator have been recently started, or if treatments with drugs targeting the symptoms of interest have been changed recently, a stable clinical status (according to treating physician) must be achieved for at least 2 months before N-of-1 therapy plan	•Epilepsy surgery less than 3 months prior to start of N-of-1 strategy •Rapidly progressive disease •Contra-indications to the precision therapy of interest according to the summary of product characteristics •Interactions of treatment of interest with other concomitant treatments, which cannot be monitored or mitigated with routine measures

**Table 2:** Treatments suitable for N-of-1 strategies in patients with rare monogenic epilepsies

Treatment characteristics	
•*The treatments which will be used in clinical N-of-1 strategies for epilepsy include*: •Precision treatments defined as treatments shown to reverse at least partially the functional consequences of the patient’s pathogenic variant. This can be based on in vitro studies or known mechanism of action in relation to pathological effects of genetic variant (e.g. gain-or loss-of-function). The treatments can be: •Medication licensed for a different indication or age group used off-label t •Substances registered as supplements (not approved as medicines) used with a therapeutic intent All treatments must have a positive risk benefit analysis as determined by multidisciplinary team of experts (including checking whether the proposed precision treatment could plausibly restore functional consequences of the genetic variant)	

Treatment features suitable for N-of-1 strategies	Treatment features unsuitable for N-of-1 strategies
•Treatments with rapid onset of action (e.g. < 2–4 weeks) and rapidly reversible effects •Potential risks deemed acceptable considering potential benefit according to multidisciplinary team of experts	•Recommended titration, and tapering periods longer than 4–6 weeks

### Justification to use the intended therapy

This blueprint refers to the use of precision treatments are defined here as treatments shown to at least partially reverse the functional consequences a specific gene defect. This can include ASMs, treatments licensed for a different indication or age group used off-label as precision treatments for monogenic epilepsies, and substances marketed as nutritional supplements but considered to have therapeutic potential (Table 2). A fundamental step in justification of the intended precision therapy involves knowledge on the functional consequences of a pathogenic genetic variant, therefore this point has been included in the benefit risk assessment checklist (**Appendix 2**).

Newly proposed off-label precision treatments to be implemented in N-of-1 strategies in care should be reviewed by a multidisciplinary expert panel (MEP) with extensive expertise on monogenic epilepsies and their treatments [29]. A MEP should include: clinical geneticist, who can comment on the genetic diagnosis and functional effects of a variant relevant for precision treatment selection, a clinical pharmacologist who can critically asses potential risks related to off-label treatment use, a methodological expert who can be consulted on the reliability of the N-of-1 design to assess the value of a treatment for an individual and a (paediatric) neurologist who can comment on the available evidence-base for the off-label precision treatment of interest, relevance and reliability of clinical outcomes. The members of the MEP should have considerable experience with similar clinical questions in at least one rare monogenic epilepsy, and in evidence-based medicine (e.g. trial design or assessment of quality of scientific evidence). A MEP does not substitute the local oversight required to conduct quality improvement of care projects, such as N-of-1 strategies. The MEP should review the potential benefits and risks associated with the off-label treatment. The MEP can determine the acceptability of treatment and provide any advice on cautionary measures (e.g. dosing schedule, use in specific sub-groups of patients, efficacy and safety assessment tools) and other aspects of the study design, such as duration of treatment and follow-up. For this purpose, the MEP should use a structured benefit-risk assessment methodology, such as an adapted version of the Benefit-Risk Assessment of Off-label Drug Use in Children (BRAvO) framework [36] (**Appendix 2**). In Europe, a multidisciplinary team composed of members of the ERN EpiCARE or European Consortium in Epilepsy Trials (ECET), with extensive expertise in rare monogenic epilepsies, has agreed to be consulted as a MEP.

### Feasibility of a N-of-1 strategy

An N-of-1 strategy may be considered an attractive approach to determine the effectiveness of a therapy for an individual. However, it should be applied only when it is feasible to answer the clinical questions of interest (Table 3) [37]. An N-of-1 strategy is only indicated in cases of clinical equipoise, and the patient and/or caregiver must be interested in following the treatment plan as outlined and contribute to define the outcomes of interest. Box 3 highlights the steps the clinician must consider to assess whether an N-of-1 therapy plan would be indicated and feasible.

**Table 3:** Questions to determine whether an N-of-1 strategy would be feasible in clinical practice

Questions to determine whether an N-of-1 strategy would be feasible in clinical practice. Adapted from Guyatt et al. 1988 [37]
*Is the N-of-1 strategy indicated for this patient?* •Is the effectiveness of treatment in doubt (i.e., do equipoise conditions apply)? •Will the treatment, if effective, be long term? •Is the patient eager to collaborate in the N-of-1 strategy and to contribute to its design (e.g., by assisting in defining outcomes)?
*Is an N-of-1 strategy feasible in this patient?* •Does the treatment have a rapid onset of action, taking any need for titration into account? •Is there sufficient information to select the target dose? •Does the treatment stop acting soon after being discontinued? If down titration is needed, can it be tapered in less than 6 weeks? Are withdrawal symptoms expected, and if so can their confounding effect be removed by a feasible wash-out period? •Is an optimal duration of treatment feasible to assess efficacy and tolerability? •Can clinically relevant effects be measured? •Can sensible criteria for stopping the trial be established?
*Is the N-of-1 strategy feasible in the clinical practice setting?* •Is there a pharmacist/clinical pharmacologist who can assist in the design/implementation of the N-of-1 strategy plan? •Are pre-defined strategies for interpreting the data in place? •Is a multidisciplinary expert panel in place to assess potential risks and benefits of the N-of-1 strategy? •If a double-blind design is in place, is an unblinded local quality improvement of care review board accessible for consultation regarding critical treatment decisions?

**Box 3:**
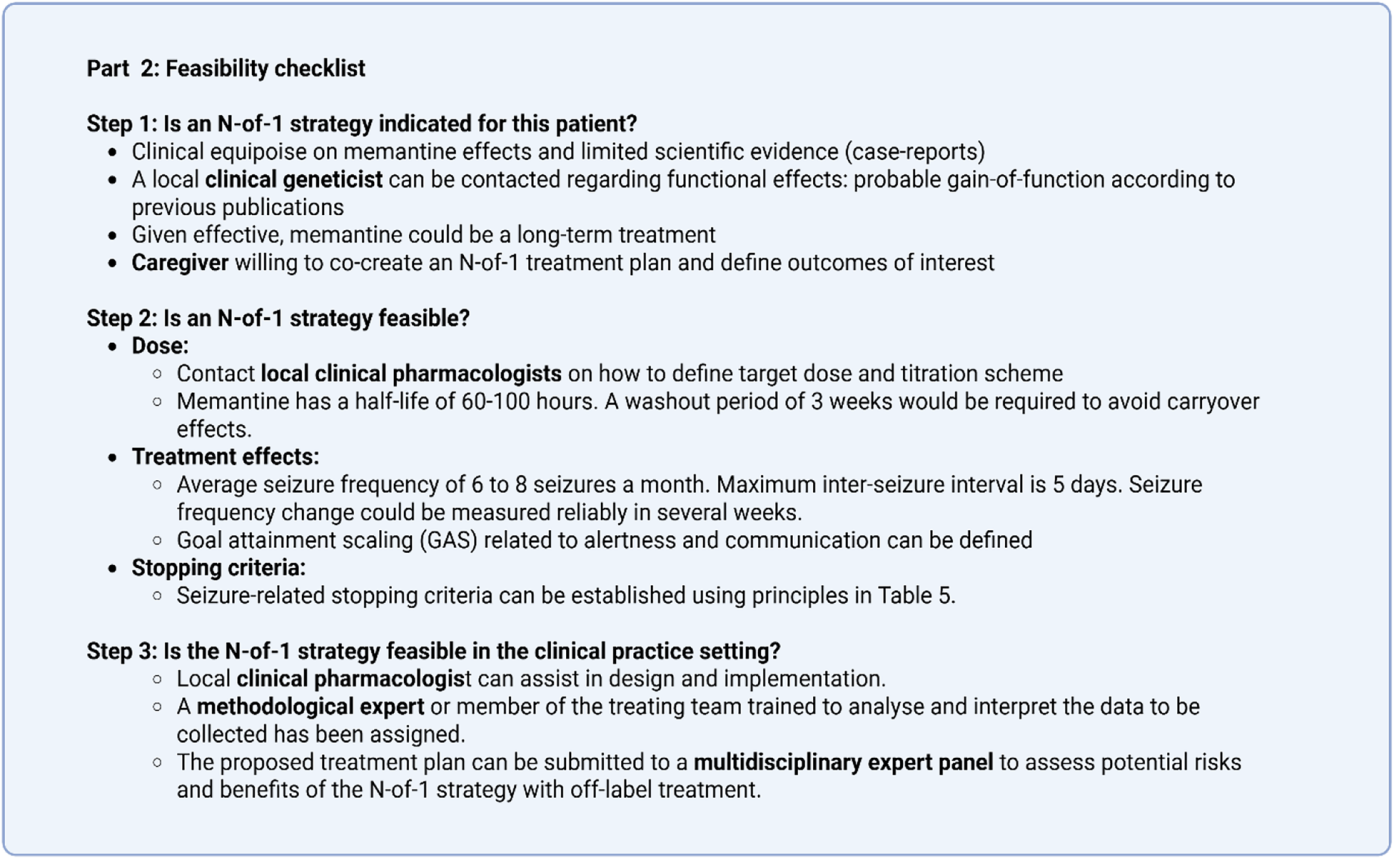
Feasibility checklist to be completed by treating clinician and local multidisciplinary support team. Aligns with checklist given in Table 3. Per step role of professionals or patient/caregiver highlighted in bold

The duration of an N-of-1 strategy affects patient adherence and logistical feasibility (Table 3 and Appendix 3, Part A). Box 4 depicts an N-of-1 therapy plan as proposed by the treating team. The characteristics of the treatment and the time needed to reliably measure the outcome of interest define the minimal period duration. Treatments with a long half-life or requiring a prolonged titration period are generally unsuitable for N-of-1 strategies (Table 2 and Appendix 3, Part A). For frequency-based outcomes, such as seizure frequency, a minimum of three times the longest interval between events can be applied as a rule of thumb to determine the period duration. Consequently, a prolonged assessment period would be required for a seizure frequency of, for example, one seizure every two or more weeks, to be able to exclude that any apparent clinical improvement is simply due to random fluctuation in seizure frequency. The duration of washout periods between treatments also needs to take into account potential carryover-effects, including the possibility of other confounders such as withdrawal seizures or other withdrawal manifestations. Gradual dose tapering may reduce, but not necessarily eliminate, the risk of withdrawal symptoms. Box 4 shows the selected period and washout duration, as well as, the individualized outcomes of interest for an individual with a monogenic epilepsy who could be treated off-label with memantine. Period duration is defined based on treatment characteristics (half-life) and seizure frequency changes as specified in Box 3

Importantly, the feasibility of an N-of-1 strategy in clinical practice is dependent upon the physician identifying other health professionals who are willing to provide the required multidisciplinary support locally as part of the treating team [37] (Table 3; Box 3). A pharmacist/clinical pharmacologist can provide support in aspects related to design (including safety and tolerability issues, relevant medication interactions, dosage and monitoring of serum drug concentration), the preparation of a placebo and the blinding and computing of a randomisation scheme. A clinical geneticist must involve in interpreting the findings of genetic testing and comment on functional effects of the genetic variant. Finally, the treating team should include a member trained in data collection and analysis, such as statistician, to prepare a pre-defined data analysis plan and advice on relevant design issues. The treating physician and the local multidisciplinary support team complete the feasibility and benefit-risk assessment checklist for the proposed N-of-1 therapy plan (Appendix 2) and submit this to the independent MEP for review (Box 5). Box 6 highlights steps to be taken by the local healthcare team to run the N-of-1 therapy plan.

### Outcome measures

A challenge which clinicians recognise from their own clinical practice is obtaining reliable measures of symptom improvement, particularly in young children and in individuals with multiple comorbidities and intellectual disability. When implementing an N-of-1 strategy, it is recommended to include a generic or disease-specific outcome measurement, a physician-reported and patient (caregiver)-reported clinical global impression of change [38, 39] and other applicable individualised outcomes.

The selected outcome should be suitable to detect changes occurring over short periods of time (≤ 3 months) and applicable in the outpatient clinic setting or from home (Table 4). Appendix 3 provides an overview of the principles relevant to selection of outcome measurements that can be used in N-of-1 strategies for monogenic epilepsies. A set of recent guidance documents have outlined several recommended tools to assess major epilepsy-related comorbidities (e,g, behaviour, sleep problems, quality of life, mood disorders) [40, 41], but some of these tools are not suitable for N-of-1 strategies in monogenic epilepsies. In N-of-1 strategies, attention should be paid to the minimum interval between sequential measurements to limit learning/practice effects. For example, the Aberrant Behavioural Checklist (ABC) is recommended for use at intervals of 4 weeks, whereas other behavioural scores require a 6-month interval [42] (Appendix 3). Finally, in the case of developmental and epileptic encephalopathies (DEEs), commonly used scores may exhibit floor and ceiling effects [43], or may not have been previously validated for a specific phenotype [44]. Individualised scores, such as the Goal Attainment Scaling (GAS), may be complementary to standardised scores in capturing meaningful change in the individual’s symptoms and level of functioning [45, 46]. In tailoring the evaluation of treatment effectiveness, there is scope for involving individual patients in the selection of the outcome measures that they personally find most relevant to their quality of life, using existing generic or disease-specific tools.

**Table 4:** Main principles for selection of non-seizure outcomes

*General characteristics*
Change in the outcome of interest should be reliably measurable over short periods of time Score or test used for assessment should retain validity when repeated at short intervals
Assessment methods (e.g., scales, or other measures) should be adequate to detect clinically meaningful changes in the outcome of interest in patients with comparable phenotypes
*Outcome measurements to be included*
One standardised measure of quality of life or daily life functioning suited for clinical use [40, 41, 78]
Adverse effects, monitored according to treatment characteristics (as in clinical care)
Clinician-reported global impression of change (e.g. CGI/S) [38]
One personalised measure of main goal to be achieved in daily life functioning, e.g. goal attainment scaling [46, 79, 80]
Outcomes related to comorbidities (behaviour, motor function, sleep, anxiety) [40, 41]

**Box 4:**
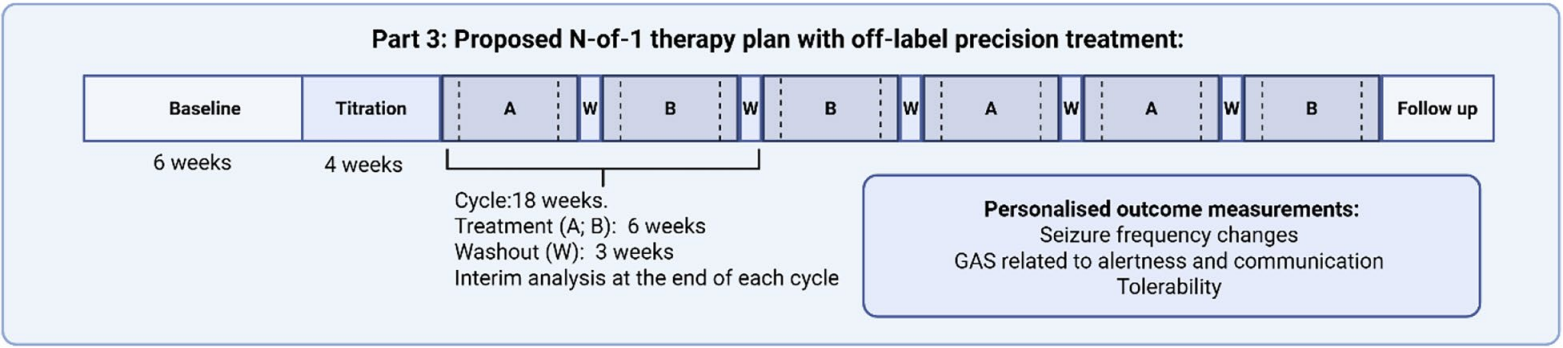
Proposed N-of-1 therapy plan with off-label precision treatment

**Box 5:**
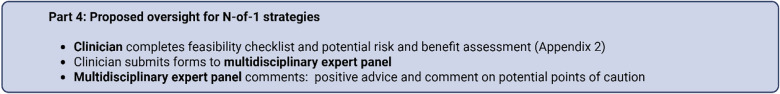
Proposed oversight for N-of-1 strategies with off-label precision treatments

**Box 6:**
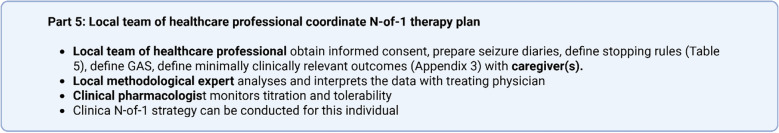
Local team of healthcare professionals coordinate N-of-1 therapy plan

### Sample size

Sample size in an N-of-1 protocol refers to a) number of measurements per period and b) number of alternating periods [13, 47, 48]. Therefore, the duration of periods per cycle as well as the number of cycles required should be determined taking into consideration the frequency of a particular measurement (Appendix 3). For non-seizure outcomes (e.g. quality of life scores, behavioural scores), a minimum of one measurement per period would be required. However, particularly when the measurement is known to be subject to significant test–retest variability, one measurement per period would require a large effect size or several periods to improve confidence in the estimated treatment effect. For seizure outcomes, individuals with frequent (daily) seizures could have multiple seizure frequency estimates (for example, median number of seizures per day over 1 week period) determined over a 4- to 6-week treatment period and therefore require fewer treatment cycles to determine treatment effects with reasonable certainty.

### Randomisation

In N-of-1 strategies, the order in which the individual receives the treatment of interest and the comparator (alternative treatment or placebo or no additional treatment, i.e. care as usual) should be randomised. The intervention of interest and the comparator(s) should be applied sequentially, in two or more cycles (Fig. 1). The aim of within-patient order-randomisation is to minimize the impact of time-dependent confounders [49]. N-of-1 strategies as outlined in this blueprint use counterbalanced blocked randomisation per cycle to ensure that equal number of periods with control and intervention are followed.

### Placebo versus active comparator and blinding

In N-of-1 strategies all patients receive conventional ASM throughout the crossover therapy plan, comparator (such as placebo) and precision treatment are adjunctive therapies (Fig. 1). Ideally, placebo should be used as comparator unless there is expectation (plausible, for some therapies specifically targeting the pathogenic gene defect) that the precision treatment being tested has superior efficacy to a conventional ASM acting by other mechanisms. Should no difference be found between treatments, data interpretation can be challenging as it may be difficult to determine whether the two treatments were equally effective or equally ineffective in that individual. The use of placebo is generally preferable to obtain an accurate estimate of the treatment’s actual efficacy and tolerability. In N-of-1 strategies in clinical care, however, use of placebo may be unfeasible due to financial and logistical challenges [50]. Whenever possible, it is advisable to seek funding for incorporation of placebo as comparator. When performing double-blind clinical N-of-1 strategies, the treating physician is blinded to treatment allocation, which could be undesirable in certain situations, such as the evaluation potential serious adverse reactions: under these conditions, an unblinded local quality improvement of care review board should be accessible for consultation regarding treatment decisions, including the application of individualised stopping rules (see section on Safety). N-of-1 strategies using open-label treatments may be partially affected by placebo and nocebo effects similar to conventional care. Should double-blinding be logistically unfeasible, the blinding of individuals responsible for assessing outcomes including seizure diary data, (video-) electroencephalography (EEG) records, neurodevelopmental tests, or behavioural scores can provide some protection from observer-related bias.

### Statistical analysis

Visual analysis of graphically tabulated results (e.g. seizure diaries) should be supported by formal statistical analysis across the crossover periods. Statistical analysis should account for autocorrelation, carryover effects and within-patient variance, which can be performed using either Bayesian or frequentist analysis methods. While both frequentist and Bayesian methods have been used, Bayesian analysis provides significant advantages by providing probabilistic estimates of the outcome of interest rather than by simply rejecting the null hypothesis, such as the *p* value computed by frequentist analysis. The use of probabilistic estimates is particularly suitable for adaptive N-of-1 strategy designs and can optimise use of scarcely available data [51].

Statistical methods can also be used to refine the N-of-1 strategy design in several ways, such as minimising duration of exposure to ineffective treatments or placebo, optimise allocation to treatments, and the use of available data. Period duration can be adjusted based on seizure frequency and estimated effect sizes through a simulation analysis [52] or using a time-to-event design. To our knowledge, no examples of the latter within the context of N-of-1 strategies have been published for epilepsy. Similarly, exposure to ineffective treatments/placebo can be reduced by performing an interim analysis at the end of each cycle [53] or a continuous outcome analysis for an individual and terminating the N-of-1 therapy plan as soon as sufficient evidence of treatment efficacy (or lack thereof) has been achieved. N-of-1 designs can also use adaptive randomisation techniques to include the most effective treatments in the subsequent cycles [12, 54–56]. For example, an N-of-1 strategy comparing three active licensed treatments could include a cycle with all three treatments and subsequent cycles with the two treatments that showed the most improvement in the outcome of interest or had the best tolerability profile for the individual patient [55]. This can be done using both Bayesian and frequentist approaches. Bayesian analysis allows use of existing knowledge generated by the results of previous patients to be incorporated in the analysis to improve the estimates for individual patients [57]. Appendix 3 discusses the rationale for statistical analyses in N-of-1 strategies in clinical care. The aim of these strategies is to assess the value of a treatment for the individual and not to combine results at group-level. Research endeavours aimed at creating generalisable knowledge could benefit from a meta-analysis on retrospective N-of-1 strategy results, considering heterogeneity in individual approaches [58, 59].

### Safety

The use of alternating treatments cycles in N-of-1 strategies can raise safety concerns related to long-term exposure to ineffective treatments, or the temporary withholding of alternative beneficial treatments. However, as discussed in the previous section, an N-of-1 strategy does not necessarily require a fixed number of cycles to be completed. At the end of each cycle, an interim assessment can be performed to determine whether continuing the N-of-1 treatment plan is justified (Fig. 1). An interim assessment plan involves consideration of desired/undesired outcomes for the individual and ‘stopping rules’, which define (a) lack of efficacy, (b) unacceptable low tolerability, (c) worsening of symptoms, which cannot be explained by natural fluctuation; (d) overwhelming evidence of benefit from one of the treatments being assessed. The ‘stopping rules’ can be applied anytime during the N-of-1 therapy plan and should define (a) desired outcomes (e.g., a predefined seizure-free period for patients with a long history of daily seizures, or a predefined major reduction in seizure frequency from baseline); (b) undesirable outcomes (e.g. pre-defined increase in seizure frequency, severity or other symptoms; (c) unacceptable adverse events. Definition of desirable and undesirable outcomes should involve an *a priori* specification of a minimum duration of the assessment period(s).

‘Stopping rules’ should be defined by taking into consideration the probability of the observed outcome being causally related to the treatment in question (magnitude of change greater than by chance). Depending on baseline seizure frequency and cycle duration, one or two cycles may be sufficient to assess seizure outcomes. Statistical modelling can be used to define the ‘effectiveness threshold’ required to establish that a minimally clinically relevant effect has been obtained [53]. Table 5 demonstrates ‘stopping rules’, which can be applied at the end of each cycle or at any time during the N-of-1 therapy plan, as well as compliance assessment checks to be performed at each interim assessment.

**Table 5:** Interim assessment and stopping rules

*Effectiveness-related stopping rules: *
Individualised effectiveness-related stopping rules should be defined by patient, caregiver and physician considering in particular scenarios that fall outside of the patient’s natural course of diseases (without aggravating factors). Examples of scenarios that should be considered in defining stopping rules include: •Increase in seizure frequency according to baseline seizure frequency and seizures types (e.g. 50% increase in frequency) •Appearance or aggravation of clusters of seizures •Change in seizure manifestations, seizure types •Changes in seizure severity (ictal duration, post-ictal period) •Episode of status epilepticus which falls outside the typical course of disease, seizure types, and response to rescue medication •Unequivocal effectiveness may also be a reason for early termination. If the patient reached the pre-defined unequivocal reduction in seizure frequency (or improvement in other outcomes), the crossover therapy plan can be terminated and a follow-up to assess effectiveness of continuation of treatment over time is advised
*Adverse effects related stopping rules:*
•Grade 3 adverse effects (including seizure aggravation) are severely disabling adverse effects which are not immediately life-threatening [81]. Grade 4 adverse effects (including seizure aggravation) are life-threatening. In both cases, the treating physician and patient may decide whether to stop or pause and continue the N-of-1 therapy plan as would be done in conventional clinical care. If there are specific concerns, the MEP or the local quality improvement of care review board can be contacted to define relation with N-of-1 therapy plan
*Adherence assessment:*
•Interruption of one or more concomitant ASMs (e.g., due to side-effects of the ASM) for a period considered relevant could lead to temporary aggravation and may justify adding an extra cycle
•Unreliable reporting in seizure diary (less than 80% of days per period reported), would justify terminating the N-of-1 therapy plan
•Medication not taken consistently, as observed in blood trough levels or counted blisters/bottles without a valid justification could also be a reason to terminate the N-of-1 therapy plan
•If medication is temporarily stopped for a medical reason (e.g. intercurrent illness), an extra cycle can be added

### Data collection

In Europe, the handling of personal data is regulated by the General Data Protection Regulation (GDPR). According to this regulation, informed consent must be obtained to process and publish de-identified personal data, if applicable. Data can be collected in the patient’s electronic health record file (EHR) as part of clinical care. Seizure frequency can be recorded on paper or in a digital seizure diary (if already part of clinical care) depending on preference. In addition, patients participating in N-of-1 therapy plan in clinical care can be asked to provide consent to collect and analyse their de-identified data as part of a learning health system in which data are regularly analysed for learning activities (quality improvement of care) and may be shared with third parties (not involved in their direct care) [60–62]. Learning activities may entail:

- Using the data of an individual to establish ‘prior probabilities’ which can be used to improve estimates for other patients.
- Determining how many individuals completed all periods of the N-of-1 strategies to inform decisions on whether certain treatment plans have been successfully implemented in care.
- Analysing data to advocate for reimbursement of treatment costs from insurance schemes.
- Using de-identified data for publication. This refers to future studies analysing clinical data retrospectively and is not part of the N-of-1 strategy in clinical care.

### Regulatory considerations

Due to their systematic design, N-of-1 strategies in clinical care have been considered a hybrid between RCTs and quality improvement of care. As such, whether these strategies should abide by legislation and ethical guidelines established for research or care has been a topic of debate [26–29]. The lack of clarity on the ethical and regulatory aspects has stalled efforts to implement N-of-1 strategies in clinical care. Recent published ethical frameworks define research N-of-1 strategies as those aimed at generating generalisable scientific knowledge on the efficacy of an investigational or licensed treatment [26, 29]. N-of-1 strategies in clinical care are aimed at improving clinical decision-making, involve measurements as done in conventional care and evaluate only licensed treatments with a documented positive benefit to risk balance [26, 29]. Research N-of-1 strategies should obtain formal IRB approval whereas N-of-1 strategies in clinical care should secure local approval as a quality improvement of care approach (Appendix 1). However, this distinction, and the respective ethical and legal oversight requirements, is subject to variation across different settings [30].

For example, in the Netherlands, the present blueprint will be used as a master protocol for quality improvement of care at UMC Utrecht. Regulations of clinical care will be adhered to in accordance with the Dutch Medical Treatment Contract Act (WGBO in the Netherlands) and Dutch Medicines Act (Geneesmiddelenwet). Written informed consent for off-label treatment and an N-of-1 therapy plan will be obtained. Individuals who may benefit from an N-of-1 therapy plan will be informed by their treating physician and receive a letter explaining their respective N-of-1 therapy plan and the option to participate in the learning health system (see ‘Data Collection’). The information will be provided in a format adjusted to their level of intellectual functioning. Signed informed consent will be obtained from the individual and/or legal representative(s).

## Discussion

N-of-1 strategies in clinical care provide a structured tool to assess the efficacy and safety of a treatment for individuals with rare epilepsies for whom potentially beneficial precision treatments are available but are scarcely used due to poor quality of available evidence and challenges translating into treatment plans for patients with phenotypic heterogeneity. Recent systematic reviews have shown that, despite their potential value, N-of-1 designs are rarely applied in epilepsy [18, 22].

The clinical N-of-1 strategies as proposed in this blueprint differ from research N-of-1 strategies in their aim to aid in improving clinical management of an individual patient. These could be applied to assess treatment tolerability and effects, identify the optimal dosage for an individual, or explore the impact of a treatment in contrast to the natural course of the disease [18]. Research N-of-1 strategies aim to generate novel scientific knowledge for a specific group of patients, subject to the regulations of research, and cannot be applied or adjusted to address the individual clinical needs. Here, we outlined ethical and logistical aspects of the distinction between clinical and research N-of-1 strategies (Appendix 1). To implement clinical n-of-1 strategies in care, local authorities should be contacted to liaise on how to implement n-of-1 strategies as quality improvement of care offering a systematic, yet personalized treatment approach.

To our knowledge, this is the first comprehensive and practical overview of how to apply N-of-1 strategies for precision therapies in individuals with rare monogenic epilepsies. As such, this blueprint represents a step to a) lay the foundation for a higher quality approach to the clinical management of these patients and b) move away from the current ‘trial and error’ approach.

The blueprint also attempts to correct for one of the major barriers to implementation of N-of-1 strategies—that is, lack of expertise and time constraints [63–65]. It does so by bringing together current guidelines and by tailoring existing knowledge on N-of-1 strategies to the context of epilepsy, thereby providing a more accessible and robust approach than currently available. This approach can ideally be implemented in the form of a master protocol.

Master protocols have emerged in response to the need to coordinate efforts to evaluate one or more treatments in multiple or single diseases following the same trial structure, particularly in the context of novel targeted therapies for (rare) genetic subtypes of a disease [66]. N-of-1 strategies performed as part of a master protocol provide an efficient and less-time consuming way to implement multiple individualised N-of-1 strategies for different patients with rare conditions within the same protocol or structure. To our knowledge, no master protocols of N-of-1 strategies in rare epilepsies have been published. However, N-of-1 trials with several different treatments for one disease have been proposed in hypertension and neuropathic pain [54, 67, 68]. A master protocol for N-of-1 strategies for patients with different rare monogenic epilepsies would involve developing a protocol structure which allows conducting several sub-studies to assess the value of different treatments designed on the basis of common premises.

We acknowledge that treatment plans based on this blueprint may have methodological limitations. Some N-of-1 strategies in clinical care may have to be unblinded due to financial or logistical constraints, and therefore results could be affected by patient and observer bias. However, regression to the mean, which is a major contributor to the clinical improvement associated with placebo treatment in parallel-group trials [20, 69], is unlikely to play a major role in N-of-1 strategies due to the randomised crossover design and the use of repeated treatment cycles. The minimum period duration suggested in this blueprint is based on the longest inter-seizure interval, as recommended in N-of-1 trial manuals [37, 49]. However, future N-of-1 strategies should optimize trial duration by statistical modelling of previously observed fluctuations in seizure cycles [18, 21]. In any case, N-of-1 strategies are not generally feasible for patients with low seizure frequency [70], unless clinically meaningful outcomes other than seizure frequency can be targeted. A drawback with some outcomes is that only one measurement per period may be justified, which can limit the robustness of the statistical analysis unless the effect size is large and rapidly reversible upon discontinuation of treatment. Despite these limitations N-of-1 strategies, with their associated repeated treatment exposures and structured assessments, represent improvement over the empirical ‘trial and error’ approach.

The toolbox provided in this blueprint (Appendix 3) recommends on guiding principles to select non-seizure outcomes for N-of-1 strategies which can be reliably measured in short periods of time. For patients with complex and drug-resistant epilepsies, comorbidties, such as behaviour, motor function and communication, may also have an important impact in daily life functioning. For certain DEEs, meaningful clinical measurements have been validated to ensure relevance of outcomes in clinical trials [43, 44]. Studies assessing minimally clinical relevance, and floor and ceiling effects of non-seizure outcomes could aid in selecting outcomes for clinical N-of-1 strategies. Similarly, the clinical N-of-1 strategy principles outlined in this blueprint can also be applied in the design of N-of-1 strategies for other rare conditions, given measurable symptoms can be monitored reliably in short periods of time. The implementation of N-of-1 strategies for precision treatments in monogenic epilepsies needs to address challenges identified in the use of precision treatments in other clinical care settings [5, 23, 24]. One challenge relates to the lack of consensus on how precision treatments should be defined [24, 71]. For consistency, we suggest the use the six-tier definition proposed by Byrne and colleagues [71]. Lack of information on the functional impact of many pathogenic gene variants and absence of simple and reliable models to predict such impact may hinder the rational selection of a precision treatment for an individual [23]. When going through the feasibility checklist for N-of-1 strategies (Appendix 2), we advise consulting with scientists with specialised knowledge of the relevant gene and possibly conducting functional tests in the laboratory. Public gene portals, such as the GRIN portal [72] which include results of functional tests, hold promise in accelerating rational use of precision treatments. Finally, lack of reimbursement for off-label precision treatments can be a major hurdle to their uptake in clinical care [23]. To address this issue, clinicians may apply for local funding for an N-of-1 driven care. For an individual patient, the results of the N-of-1 strategy could be used to apply for reimbursement for chronic use from public or private insurance schemes. Evaluation of data from multiple N-of-1 strategies (group-level results) could also be used to apply for licensing and reimbursement procedures [73].

Another important challenge of the use of off-label precision treatments for rare epilepsies in the context of N-of-1 strategies is the lack of guidance for dose selection. Typically, available data for precision therapies in rare epilepsies are limited to a few case reports, in which dose was adjusted according to clinical response and with typically large dose variation across individuals [74–77]. As a result, rational preselection of a fixed dose remains challenging. The optimal dose for one individual is unknown. Using each crossover period to optimize dose based on clinical response will prolong the trial beyond feasibility. One potential solution is to have an open label dose-exploration phase preceding the N-of-1 crossover phase: once the optimal dose is identified that dose will become the target dose to be given in the crossover phase. This approach has drawbacks, because the patient may learn how to recognise the active treatment, resulting in unblinding during the crossover phase.

The current blueprint for N-of-1 strategies has been developed to focus on off-label precision treatments for monogenic epilepsies due to the ubiquitous encounters with clinical equipoise; however, clinical N-of-1 strategies may also be applied in other situations of uncertainty regarding the value of a treatment for an individual. In clinical practice, the selection of ASM is based on seizure semiology or syndrome being treated, comorbidities and according to drug tolerability and interactions with comedication. However, despite available evidence from RCTs and existing treatment guidelines, the therapeutic effect and tolerability of an ASM may differ per patient. When there is uncertainty regarding the efficacy of a specific treatment/ASM for an individual patient, a clinical N-of-1 strategy comparing two active ASMs may be useful and justified to assess treatment effects in a more systematic and objective manner. The current blueprint could be adapted to define the characteristics of patients and equipoise situations in which an N-of-1 therapy plan with conventional ASMs in patients with epilepsy could be applied.

This blueprint should be applied by taking into consideration oversight and regulatory requirements specific to the region or country in which N-of-1 strategies are applied. Despite efforts to establish international N-of-1 protocols, country-specific requirements continue to demand tailored implementation, and efforts to accelerate use of N-of-1 strategies may encounter local logistical and financial constraints (Appendix 1). In the United Kingdom (UK), the National Institute for Health Research commissioned a project for the development of generalisable methodology for N-of-1 trials for low volume treatments (as is the case with rare monogenic epilepsies) [63]. The project aims at providing footholds for physicians and researchers to design and conduct N-of-1 trials geared at informing decisions about clinical care. In the UK, a treatment plan involving predefined treatment allocation requires regulatory approval as research, and therefore the procedures to implement the aforementioned initiative may differ from the approach exemplified in the Netherlands. An example of a locally developed N-of-1 approach in clinical care which is specific for epilepsy is a protocol proposed in Australia, using resources funded by the Victorian Department of Health and Human Services [52]. In short, it is always advisable to consult the local quality improvement of care team to determine how to adapt and implement this blueprint, preferably within the format of a master protocol.

## Conclusions

We propose that the guiding principles described in this blueprint will aid clinicians in designing and conducting N-of-1 strategies in clinical care, thereby improving rational application of off-label precision treatments for monogenic epilepsies. This blueprint can be implemented in different centres according to local regulatory conditions and expertise.

## Supplementary Information

Supplementary material 1

Supplementary material 2

Supplementary material 3

## Data Availability

Data sharing is not applicable to this article as no datasets were generated or analysed during the current study.

## Abbreviations

PINPOINT: Study acronym stands for: Precision Treatments In MoNogenic EPilepsies: Observational Registry And N-of-1 Trial Recommendations
ASM: Antiseizure medication
RCT: Randomized controlled trial
ERN EpiCARE: European Reference Network for Rare and Complex Epilepsies
CCMO: Dutch Central Committee on Research Involving Human Subjects
SPIRIT: Standard Protocol Items: Recommendations for Interventional Trials
SPENT: SPIRIT extension for N-of-1 trials checklist 2019
CONSORT: Consolidated reporting items for trials
CENT: CONSORT extension for N-of-1 trials
UMC Utrecht: University Medical Centre Utrecht
MEP: Multidisciplinary expert panel
BRAvO: Benefit-Risk Assessment of Off-label drug use in children framework
ECET: European Consortium in Epilepsy Trials
ACMG: American College of Medical Genetics
ABC: Aberrant Behavioural Checklist
GAS: Goal attainment scaling
EEG: Electroencephalography
GDPR: General Data Protection Regulation
EHR: Electronic health record file
UK: United Kingdom
ePAGs: EpiCARE European Patient Advocacy Groups
