# Supplementary material for: Blueprint for clinical N-of-1 strategies with off-label precision treatments in monogenic epilepsies
Source: Orphanet J Rare Dis. 2025 Jun 16;20:309. doi: 10.1186/s13023-025-03750-z (PMC12172224; doi:10.1186/s13023-025-03750-z)
Supplement: Supplementary file 1 — Supplementary material 1 [file 13023_2025_3750_MOESM1_ESM.docx]

**Appendix 1: Ethical and legal review considerations of N-of-1 strategies**

N-of-1 strategies originally emerged as forms of improving treatment selection in cases of clinical equipoise (1,2) and have only recently been re-discovered as an emerging source of high-quality evidence, which can offer an alternative for randomized controlled trials (RCTs) in small patient populations or populations with heterogeneity of treatment effects(3,4). Due to their systematic design and focus on the outcome in a single individual, N-of-1 strategies have been considered a hybrid between scientific research and quality improvement of care. Therefore, whether N-of-1 strategies should abide to legislation and ethical guidelines established for research or care has been a matter of debate. The lack of clarity on the ethical and regulatory aspects of N-of-1 studies has stalled efforts to conduct N-of-1 studies, particularly in the clinical context.

In 1995, Irwig and colleagues published the first proposal for ethical assessment of N-of-1 strategies in the Lancet (5). They stated that N-of-1 strategies should be seen as a tool to aid in clinical decision making which can minimize the bias in a physician’s judgement of “treatment response” and would not require Institutional Review Board (IRB) approval despite including aspects of research in their design. Recently, two comprehensive frameworks to assess when N-of-1 strategies should be subject to IRB approval as scientific research have been published (6,7). An important distinction on whether N-of-1 strategies should be subject to the regulations of research or care have been made based on the definition of medical scientific research: Is the aim of the N-of-1 strategy to obtain, new, generalizable knowledge that answers a question that extends beyond the participant? Or is the intention simply to improve clinical management in an individual? The former refers to a the aim of a research N-of-1 strategy and the latter to the aims of a clinical N-of-1 strategy. (6,7).

Different forms of oversight apply for different N-of-1 strategies. N-of-1 strategies in clinical care are aimed at assessing the value of a treatment for an individual patient, limited to treatments with an acceptable risk-benefit ratio (as assessed by an external multidisciplinary expert panel (MEP), and involving procedures normally followed in standard patient care (6). A N-of-1 strategy in clinical care is subject to oversight from a MEP and/or local hospital quality assurance team (6). This differs from research N-of-1 strategies, which aim at creating generalizable scientific knowledge, with additional procedures and measurements often involved to answer a scientific question (6). A research N-of-1 strategy is subject to mandatory IRB review as interventional medical research following the European Union Clinical Trial Regulation (EU CTR) 536/2014 and local research procedures.

If an N-of-1 strategy in clinical care would need to undergo a mandatory IRB review as interventional medical scientific research according to the EU CTR, this would require substantial efforts and costs related to study medication, monitoring procedures, additional hospital visits and extensive documentation (8). Moreover, this would cause harmful delays due to the lengthy administrative procedures involved. This scenario has consequences for the care of patients with rare epilepsies with unmet medical needs (summarized in **Table S1**) who could benefit from potential off-label treatments and may be unfeasible and disproportionate for one or two patients of this rare population. As a result, physicians may opt for the trial-and-error approach with its associated drawbacks, or decide not to put in the needed extra effort for a potentially beneficial treatment. The administrative load may also cause delays in clinical care, which could be a problem for patients with severe epilepsies who for whom waiting 30-60 days before implementing a treatment plan may be harmful. Indirectly, other clinical processes may also be delayed.

Should N-of-1 strategies in clinical care have to abide by research regulations, selection of patients and treatments for these strategies will likely shift towards the most prevalent epilepsy syndromes and treatments with better marketing potential, and less towards patients with more rare epilepsies and therapies that are less appealing for future commercial application. The danger of disincentivizing N-of-1 strategies in the clinical care of patients with rare and complex epilepsies requiring a highly individualized approach is real. In order to implement N-of-1 strategies readily as improvement of care, criteria for applying responsible N-of-1 strategies in clinical care and appropriate oversight procedures need to be clearly defined, as proposed in the current Blueprint document.

**Table S1:** Consequences of applying regulation of medical research to N-of-1 strategies in the clinical care of patients with rare epilepsies

| ***N-of-1 strategy as medical research*** | ***Challenges*** | ***Consequence for rare epilepsy patients*** |
| --- | --- | --- |
| *Protocol with strictly defined inclusion/exclusion criteria* | Prioritizes external validity of results over the use of a more personalized design | May exclude patients with complex phenotypes (multiple comorbidities) |
|  | Administrative burden of preparing treatment- or patient-specific protocols when the design is adjusted to patients not fulfilling initial eligibility criteria. | Conventional trial-and-error approaches will be preferred to well-designed N-of-1 strategies in clinical care. |
| *Submission to IRB as medical scientific research* | Requires extensive administrative burden (including approval, notifications). | Unfeasible for an individual patient requiring treatment soon. Can lead to major delays or disproportionate administrative burden for individuals with rare epilepsies.  Physicians may opt to not try the treatment or follow the ‘trial and error’ approach. |
| *Research-related procedures* | Additional monitoring required to comply with medical research regulations | Additional costs and manpower required to complete documentation. |
|  | Pharmacy costs if considered medical scientific research requiring use of investigation medical product (IMP) | Application to external funding would be required. Additional delays to obtain funding and obtain IMP. |

**References**

1. Guyatt G, Sackett D, Taylor D, Ghong J, Roberts R, Pugsley S. Determining Optimal Therapy — Randomized Trials in Individual Patients. New England Journal of Medicine. 1986;315(12):767–8.

2. Mirza RD, Punja S, Vohra S, Guyatt G. The history and development of N-of-1 trials. J R Soc Med. 2017;110(8):330–40.

3. Cornu C, Kassai B, Fisch R, Chiron C, Alberti C, Guerrini R, et al. Experimental designs for small randomised clinical trials: An algorithm for choice. Orphanet J Rare Dis. 2013;8(1).

4. Zucker DR, Ruthazer R, Schmid CH. Individual (N-of-1) trials can be combined to give population comparative treatment effect estimates: Methodologic considerations. J Clin Epidemiol. 2010 Dec;63(12):1312–23.

5. Irwig, L., Glasziou, P., & March L. Ethics n-of-1 trials. Lancet. 1995;345:469.

6. Defelippe VM, J.M.W. van Thiel G, Otte WM, Schutgens REG, Stunnenberg B, Cross HJ, et al. Toward responsible clinical n-of-1 strategies for rare diseases. Drug Discov Today [Internet]. 2023;28(10):103688. Available from: https://doi.org/10.1016/j.drudis.2023.103688

7. Stunnenberg BC, Deinum J, Nijenhuis T, Huysmans F, van der Wilt GJ, van Engelen BGM, et al. N-of-1 Trials: Evidence-Based Clinical Care or Medical Research that Requires IRB Approval? A Practical Flowchart Based on an Ethical Framework. Healthcare. 2020;8(1):49.

8. Joly Y, So D, Osien G, Crimi L, Bobrow M, Chalmers D, et al. A decision tool to guide the ethics review of a challenging breed of emerging genomic projects. European Journal of Human Genetics [Internet]. 2016;24(8):1099–103. Available from: http://dx.doi.org/10.1038/ejhg.2015.279
