# Supplementary material for: Blueprint for clinical N-of-1 strategies with off-label precision treatments in monogenic epilepsies
Source: Orphanet J Rare Dis. 2025 Jun 16;20:309. doi: 10.1186/s13023-025-03750-z (PMC12172224; doi:10.1186/s13023-025-03750-z)
Supplement: Supplementary file 2 — Supplementary material 2 [file 13023_2025_3750_MOESM2_ESM.docx]

**Appendix 2: Assessment of N-of-1 strategies in clinical care by a multidisciplinary expert panel**

The physician should first complete the checklist below as a quick assessment on whether an N-of-1 strategy is feasible for the condition and the treatment of interest (1). If an N-of-1 strategy is considered feasible, then the physician completes the risk-benefit analysis form for the (off-label) treatment. Both forms should be assessed by the multidisciplinary expert panel (MEP) prior to implementation of the N-of-1 strategy, according to our proposed oversight scheme for responsible N-of-1 strategies in clinical care (**Appendix 1**) (2).

1. **Checklist to assess feasibility of proposed N-of-1 strategy for off-label precision therapies in rare and complex epilepsies**

**Step 1: Patient characteristics**

- Drug resistant epilepsy: tried two or more adequately chosen and dosed antiseizure medications for an adequate period of time.
- Clinical equipoise applicable to the treatment to be tested

**Step 2: Characteristics of genetic variant (for monogenic epilepsies)**

- Variant identified: Class IV or V genetic variant according to American Medical College of Genetics and Genomics
- Functional analysis not required
  - Presumed loss of function due to truncation
  - Variant previously described as gain- or loss-of-function in literature or gene-portal and functional effects described. Add reference (PMID): ______________________________
  - Functional effects clearly predicted based on phenotype
  - Functional effects will be tested. Add method and contact of lab or investigator equipped to complete functional analysis: ________________________________

**Step 3: Off-label treatment characteristics (provided as add-on treatment to standard care)**

- Treatment of interest has rapidly reversible effects
- Treatment titration and tapering phase <4 weeks each
- Treatment up titration ~4-6 weeks but the comparator is another active treatment for which target dose can be rapidly achieved (limiting time exposure to ineffective doses of potentially beneficial treatments)
- Treatment is expected to correct the functional defect(s) responsible for the individual’s epilepsy

Reason (brief) and add reference (PMID): ___________________________________________________________________________

**Step 4: Design**

- Outcome measurement of interest can be measured in relative short periods of time (<3 months)
- Team has expertise in selecting appropriate outcome measures, trial duration and statistical analysis (see Appendix 3: Toolbox for clinician)
- N-of-1 strategies will use an adjunctive-therapy design, with continuation of pre-existing antiseizure medications

**Following steps:** If all the above checklist points have been met, an N-of-1 strategy may be feasible.

- Contact experts for additional advice on functional effects of genetic variants
- If not, all criteria are met, or you have reasons to doubts about whether an N-of-1 strategy would be suitable, also contact MEP for advice
- Complete risk-benefit assessment analysis and submit to MEP

1. **Risk-benefit analysis**

The following form has been adjusted from the BRAvO framework for benefit and risk assessment of off-label treatments-in children (3). The aim is to mitigate risks and justify the decision to use the treatment. Provide concise answers and, when possible, refer to literature or official drug documentation.

| **Drug name:**  **Dose, frequency and route of administration:** | |
| --- | --- |
| **Diagnosis (condition, severity):** | |
| 1. **Problems and Alternatives** | |
| Rationale for off-label treatment selection (reason to expect potential benefit from off-label treatment) |  |
| Licensed indication |  |
| Intended off-label indication |  |
| Is the treatment for off-label use licensed for the same age group and the same target dose for another condition? |  |
| 1. **Objectives: Efficacy** | |
| Are there relevant differences in disease phenotype, maturity of target organs or drug clearance pathways between adults and children? | Only answer if drug is not approved for the individual’s age |
| Based on the mechanism of action: is the drug likely to be effective? | *Detail reason* |
| If available, reference studies with the intended off-label use? |  |
| **Level of evidence for use in the proposed off-label indication and in patients with characteristics comparable to those of the individual for whom off-label use is proposed** | Level A1: Systematic review or meta-analysis  Level A2: Randomized controlled trials of good quality and size |
|  | Level B: Other comparative studies: RCTs of poor quality or small size, non-randomized trials, cohort studies, patient control studies, retrospective studies with sufficient size |
|  | Level C: Non-comparative trials, case reports, case series, retrospective studies of poor quality  Level D: Expert opinion, posters, or abstracts without publication of data |
| 1. **Objectives: Safety** | |
| Please specify how unacceptable safety/tolerability will be defined in the proposed N-of-1 strategy? |  |
| Are there serious adverse effects reported for this treatment?  What are the most common adverse effects reported in adults and/or children?  What are the main risk factors for adverse effects?  Are adverse effects dose-dependent? | *Refer to Summary of product characteristics and provide risk estimates as known* |
| Please summarise available safety data from use in the proposed off-label indication and in patients with clinical characteristics (age, co-morbidities, etc) comparable to those of the individual for whom off-label treatment is proposed (if available or indicate otherwise) |  |
| What measures can be implemented to prevent or minimize harm? | *Refer to laboratory assessments, precautions, stopping rules, or other measurements to identify adverse effects* |
| What risks cannot be mitigated by preventive measures? This is the residual risk |  |
| **Level of evidence for use in the proposed off-label indication and in patients with characteristics comparable to those of the individual for whom off-label use is proposed:** | Level A1: Systematic review or meta-analysis  Level A2: Randomized controlled trials of good quality and size |
|  | Level B: Other comparative studies: RCTs of poor quality or small size, non-randomized trials, cohort studies, patient control studies, retrospective studies with sufficient size |
|  | Level C: Non-comparative trials, case reports, case series, retrospective studies of poor quality  Level D: Expert opinion, posters, or abstracts without publication of data. No safety studies in the proposed off-label indication. |
| 1. **Objectives: dose** | |
| Can clinical response be predicted or monitored based on target drug concentrations in body fluids or pharmacodynamic (PD) parameters? |  |
| What pharmacokinetic (PK) data are available for individuals with clinical characteristics comparable to those of the person for whom off-label treatment is proposed? | Refer to Summary of Product Characteristics (specify, adults or children). |
| Considering available data, what dosing regimen (titration and target dose) is proposed for the person for whom off-label treatment is proposed? |  |
| Is the proposed dosing schedule consistent with that stated in the Summary of Product Characteristics for individuals with comparable characteristics (e.g. age, any relevant comorbidities)?  If the answer is no, please justify choice of dosing schedule, summarize previous data and address safety considerations |  |
| **Level of evidence available to identify the correct dose for use in the proposed off-label indication and in patients with characteristics comparable to those of the individual for whom off-label use is proposed:** | Level A: Good quality PK studies and dosing information in age-group of interest (adult, children, infant) |
|  | Level B: Good quality PK studies and dosing information in a different age group, but no expected differences based on underlying disease and developmental factors |
|  | Level C: Limited quality PK studies and dosing information in the age-group of interest or differences based on underlying disease and developmental factors are expected |
|  | Level D: Studies in which limited PK data are collected (e.g. single samples at steady state) or no PK studies in the age-group of interest |
| 1. **Objectives: Drug-drug interactions** | |
| Are clinically relevant PK or PD drug interactions known or expected to occur between the treatment of interest and any other drug/treatment that the individual is receiving? |  |
| If the answer to the above question is yes, what measures can be taken to prevent/mitigate any potential adverse effect (including loss of efficacy) resulting from these interactions? |  |
| Level of evidence delineating drug-drug interactions for use in the proposed off-label indication in patients with characteristics comparable to those for whom the off-label use is proposed (e.g. similar comorbidities, comedication): | Level A: Good quality clinical studies on drug interactions of interest and measures to mitigate potential adverse consequences |
|  | Level B: Good quality *in vitro* or other studies that allow prediction of the drug interactions of interest and measures to mitigate potential adverse consequences |
|  | Level C: Limited quality *in vitro* or other studies that allow prediction of the drug interactions of interest and measures to mitigate potential adverse consequences  Level D: No studies that allow prediction of potential drug interactions of interest and measures to mitigate adverse consequences |
| 1. **Trade-offs - *This section constitutes an explicit judgement about the potential favourable and unfavourable effects of off-label use of the treatment*** | |
| Are the expected benefits clinically relevant? |  |
| Are the residual risks (risks that cannot be mitigated) acceptable? |  |
| Do the benefits outweigh the residual risks? Or does the expected benefit outweigh the residual risks only under certain conditions (e.g. risk factors)? |  |
| 1. **Uncertainty - *This section summarizes the level of evidence and uncertainty detailed in the previous sections and clinical experience*** | |
| What is the extent of uncertainty due to the quality of the evidence (clinical studies, PK data, clinical experience)? | Level of evidence for efficacy: *e.g. Level C* |
|  | Level of evidence for safety: *e.g. Level B* |
|  | Level of evidence for right dose: *e.g. Level B* |
|  | Level of evidence for drug interactions: *e.g. Level D* |
| Level of clinical experience with off-label use of this treatment | **Level A:** Extensive experience with off-label use in this age group |
|  | **Level B:** Some experience with off-label use or prescribing for this age group for different indication |
|  | **Level C:** Experience with off-label use in a different age group and/or indication |
|  | **Level D:** Novel use, clinical experience lacking or limited |
| If the evidence is weak, why is the risk to benefit balance deemed to be acceptable for the intended use? |  |
| **Risk tolerance:**  *Multidisciplinary expert panel summarizes opinion on risk/benefit assessment of the off-label use of the treatment for patients*  *If additional information on PK parameters, dosage and other issues* *are required, the MEP will communicate with the applicants. Conflicts of interest are addressed.* | |

**References**

1. Guyatt G, Sackett D, Adachi J, Chong J, Rosenbloom D, Pharm D. A clinician’s guide for conducting randomized trials in individual patients. CMAJ. 1988;(139):497–503.

2. Defelippe VM, J.M.W. van Thiel G, Otte WM, Schutgens REG, Stunnenberg B, Cross HJ, et al. Toward responsible clinical n-of-1 strategies for rare diseases. Drug Discov Today [Internet]. 2023;28(10):103688. Available from: https://doi.org/10.1016/j.drudis.2023.103688

3. van der Zanden TM, Mooij MG, Vet NJ, Neubert A, Rascher W, Lagler FB, et al. Benefit-Risk Assessment of Off-Label Drug Use in Children: The Bravo Framework. Clin Pharmacol Ther. 2021;110(4):952–65.
