# Supplementary material for: Blueprint for clinical N-of-1 strategies with off-label precision treatments in monogenic epilepsies
Source: Orphanet J Rare Dis. 2025 Jun 16;20:309. doi: 10.1186/s13023-025-03750-z (PMC12172224; doi:10.1186/s13023-025-03750-z)
Supplement: Supplementary file 3 — Supplementary material 3 [file 13023_2025_3750_MOESM3_ESM.docx]

**Appendix 3: Toolbox to design clinical N-of-1 strategies**

This toolbox includes general principles to design N-of-1 strategies for epilepsies. The section on treatment characteristics and outcomes of interest will guide you on how to structure the N-of-1 strategy and determine when an N-of-1 strategy is not a suitable approach. The toolbox contains three parts: (A) Defining period duration (B) Outcome measurements and (C) Statistical analysis. *The lists and references provided in this toolbox are not exhaustive and serve mainly as an example and/or indication of where to obtain additional information.*

General principles to conduct responsible clinical N-of-1 strategies (stopping rules, interim assessment, oversight by a multidisciplinary expert panel (MEP), informed consent, data management) can be found in the main text of the blueprint.

1. **Define period duration and feasibility of N-of-1 strategy**

Treatment characteristics and the outcome of interest are the two main factors to decide on appropriate period duration in an N-of-1 strategy (1–3). The following considerations can be used to identify whether the treatment of interest is suitable for an N-of-1 strategy and indicate a minimum period duration for seizure frequency as outcome. Finding the lower limit of treatment cycle duration is important to prevent too lengthy trials that increase the risk of poor patient adherence and may be considered unethical given the remaining uncertainty on treatment benefit throughout the entire duration of the N-of-1 strategy.

1. **Treatment characteristics**

In order to define whether an N-of-1 strategy would be feasible with a specific therapy the following treatment characteristics should be considered:

- **Therapeutic dose:** Dose at which clinical effects (antiseizure or other symptoms) have been observed.
- **Tolerable dose:** Dose with acceptable or no side-effects. The maximum tolerable dose would be the highest dose of a drug with acceptable side-effects.
- **Titration** refers to the recommended time required to achieve an optimal dose and minimize the risk of adverse effects (4). Titration may be required at the start of each period. A titration phase could also be used initially for dose-finding purposes and the optimal dose can be restarted after each crossover with shorter or no titration depending on the influence of titration on tolerability. Treatments requiring a prolonged titration phase (>4-6 weeks) at the start of each period may lead to an excessive period duration in the N-of-1 design, which would be undesirable. Treatments requiring titration phases between 4-6 weeks could be used in an N-of-1 design if the comparator is another active treatment with potential beneficial effects for which a maintenance dose can be reached relatively rapidly. In such cases, while the N-of-1 strategy would be lengthy this may be justified by providing two treatments with expected potential benefit, with overlap between dose tapering of one treatment with titration or maintenance dose of the second treatment.
- **Tapering** refers to the period of gradual dose reduction required to minimize the risk of withdrawal seizures when stopping a treatment. Optimal duration of tapering may depend on the maintenance dose achieved and the duration of treatment, which needs to be adjusted to account for baseline seizure frequency.
- **Half-life and carryover effects:** Carryover effects refer to persisting effects of a treatment into a subsequent period when the treatment has been stopped. A minimum interval of five half-lives is required to ensure the drug has been fully eliminated, though even some effects, including withdrawal symptoms, may outlast disappearance of the drug from the body. Some drugs, e.g. vigabatrin, also have irreversible or slowly reversible actions, which can lead to prolonged carryover effects. The duration of a washout period should be calculated based on the drug’s half-life/duration of effect and integrated into the design to minimise the risk of carryover effects of one treatment into the next period (**Table 1**). If a wash-out period is undesirable, treatments can be crossed over without any treatment-free interval, but the interval expected to be associated with carryover effects after stopping the first treatment should be excluded from assessment of the effect of the second treatment.
- **Minimum time required for onset of antiseizure effects**: This is probably unknown because the minimum therapeutic dose for an individual typically has not been established. In some cases, previous studies may indicate an approximate interval after which antiseizure effects become detectable on a given dose. While the ability to detect onset of antiseizure activity is dependent on the baseline seizure frequency of the individual, available data may allow an estimate of the minimum duration required for onset of antiseizure effects or to observe these effects clinically. It is important to determine whether the desired therapeutic effects are expected to be present for several weeks over a period of maximum 3 months, otherwise the N-of-1 design may be unsuitable.

**Table 1: Treatment characteristics and minimum washout duration,** **assuming that treatment effects closely mirror the time course of plasma drug levels.**

| **Half-life** | **Minimum washout duration** | **Suitable for N-of-1 design** |
| --- | --- | --- |
| < 5 hours | 1 day | Yes |
| 6-10 hours | 2 days | Yes |
| 15-20 hours | 3-4 days | Yes |
| 30 hours | 6 days | Yes |
| 70 hours | 15 days | Yes |
| 100 hours | 21 days | Doubtful, a long-washout period could extend excessively N-of-1 strategy duration. |
| 140 hours | 29 days | No |

1. **Seizure frequency**

Ideally, period duration to capture change should be done using a statistical model considering baseline seizure frequency, longest inter-seizure interval during the baseline period and the minimally clinically relevant effect. N-of-1 trial manuals suggest using the ‘inverse rule of three’ to determine period duration in frequency-based outcomes (1,2). For example, if the longest interval between seizures during the baseline period was one week, a three-week evaluation period should be enough to have 95% certainty that at least one seizure will occur during that period, unless the intervention has been effective in reducing seizure frequency. An appropriate prospective or retrospective baseline duration should be used to define the maximum inter-seizure interval. We and others have discussed the limitations of this rule of thumb in epilepsy (5,6), however, currently there are no other readily applicable alternatives or statistical models to define period duration for frequency-based outcomes. Similarly, despite increasing number of studies having identified methods to characterize seizure cycle fluctuations (7–10), there are currently no methods available to apply this in a simple manner when defining period duration for N-of-1 strategies. Maintenance (assessment) phases longer than 6-10 weeks may prolong each cycle excessively, considering any additional time required for titration, tapering and wash-out. Application of a N-of-1 strategy would be unfeasible under these conditions.

1. **Outcome measurements of interest for N-of-1 strategies**

- Seizure outcomes
- EEG
- Quality of life (QoL)
- Behaviour
- Speech/Language
- Motor function
- Cognition – short term

1. **Seizure counts as outcomes**

The limitations of monitoring seizure outcomes, including limitations in seizure reporting (e.g. recall bias), have sparked interest in more objective measures of epileptic activity **(Table 2).** Electronic seizure diaries show better reporting precision than paper diaries (11–13) and should be preferably used.

**Table 2: Considerations for assessment of different seizure types as outcomes in N-of-1 strategies.** Adapted from Buchhalter et al. 2022 (14)

| **Seizure types** | **Limitations** | **Recommendations to measure outcome of treatment** |
| --- | --- | --- |
| Myoclonic seizures, typical absences, other  non-motor seizures, sleep related seizures | - Counting unreliable unless it is based on continuous EEG monitoring - Subtle - Lack of awareness of proxy - Impairment of patient’s consciousness | - **Sleep-related seizures:** Nightwatch or other wearable devices. - **Absences, myoclonic:** EEG recording. Seizure-free days may be a feasible measure, particularly for myoclonic seizures - **Focal non-motor impaired awareness seizures:** seizure diaries generally accepted despite recall bias |
| Several seizure types | - Patient/proxy reported, not all correlated with actual seizure events | - Confirm whether EEG correlated - Define seizure types to be monitored |
| Clusters | - Difficult to count and define | - Define cluster: several seizures occurring directly after each other or several seizures in a day - Define: frequency of clusters, severity of seizures in a cluster |
| High seizure frequency and/or several seizure types | - Reporting bias - Seizure free days may correlate better with QoL changes | - Report seizure free days (e.g. Lennox-Gastaut Syndrome) (15) |

1. **EEG outcomes**

There is an increasing interest in EEG biomarkers to be used in a structured manner in clinical care and trials (14). However, there are few EEG measures that have been validated as measures of treatment response. We recommend using EEG as outcome in cases when seizure counts based on patient/caregiver reporting are unreliable (generalised non-motor seizures) and when EEG changes have been shown to correlate with improved clinical outcomes such as cognitive development and thus can be used for therapeutic decision purposes. One example is the presence of hypsarrhythmia in infantile epileptic spasms syndrome (16–20). For epileptic encephalopathy with spike and wave activation during sleep (EE-SWAS) improvement in spike-wave index (SWI) during sleep has been to be associated with improvement in cognitive outcomes in some but not all studies (21–23).

We advise using the SCORE method of EEG reporting to facilitate interpretation (24). For measuring spike wave index (SWI) and prolonged EEG discharges, several tools are available. We recommend using PERSYST, which shows good sensitivity, specificity, and inter-rater reliability, and minimizes time required to score SWI (25,26).

1. **Quality of life**

While seizure frequency reduction has been traditionally the main focus of ASM therapy, and the endpoint of most clinical ASM trials, studies have shown that a decrease in seizure frequency alone often does not translate into improvement in quality of life (QoL) (27–29). Recent research has aimed at assessing the utility of QoL scores as a screening tool in clinical care (30–32), and defining cut-off values (or minimally clinically relevant change) to identify key issues to be addressed in clinical management (33–36). However, a minimally clinically important difference is a population-specific measure, which may not apply to patients with monogenic epilepsies as a heterogenous group (37).

Selection of a quality-of-life score in an N-of-1 strategy in clinical care should be based on the feasibility to repeat the measurement at regular intervals. To meet this objective, the scoring tool should be easy to complete (10-15 minutes) and – considering the minimum interval of time required between tests – as free as possible from learning/practice effects. In terms of psychometric qualities, the tool should have good construct validity, test-retest reliability (stability of the scores within the patient under the same conditions), and responsiveness (ability to capture clinically meaningful change).

The tables below provide an overview of several QoL questionnaires which could be suitable for n-of-1 strategies based on results of recently published systematic reviews by Mitchell at. 2024 and Crudgington et al. 2020 (31,32,38). We added the minimum interval between tests. This list is not exhaustive.

**Table 3: Selected quality of life questionnaires for paediatric epilepsy patients.**

| **Score** | **Constructs being measured** | **Applicable age / user** | **Validity** | **Test-Retest**  **Reliability** | **Minimal interval** | **Reference** |
| --- | --- | --- | --- | --- | --- | --- |
| **IPES** | 11-items  Academic achievement, participation in activities, health, relationships with family and peers, social activities, self-esteem, caregiver’s hopes for their child’s future | 2-16 years /  Proxy-reported only | Good structural validity and adequate construct validity | Doubtful, due to limited quality of studies | Not stated | (39,40) |
| **PedsQL Epilepsy Module** | 5-domains  29-items  Impact, cognitive, sleep, executive function, and mood/behaviour | 2-18 years /child self-report and  proxy-report | Good structural validity and construct validity | Adequate | 4 weeks | (34,41) |
| **QOLCE-16** | 16-items  Cognitive, emotional, social, and physical wellbeing and overall QoL | 4-18 years/ proxy-reported only | Good structural validity and construct validity | Not studies identified that measure this property | 4 weeks | (42,43) |
| **CHEQoL-25** | 25-items  Quality of life: Interpersonal/social consequences, worries and concerns, intrapersonal/emotional, secrecy and concealment, quest for normality | 6-15 years/ child self-report and proxy-report | Good structural validity and construct validity | Adequate | Not specified, performed every 4 weeks, up to 28 weeks in studies | (44,45) |

**Table 6: Selected quality of life questionnaires for adult epilepsy patients.**

| **Score** | **Domains** | **User** | **Validity** | **Reliability**  **Test-Retest** | **Minimal interval** | **Reference** |
| --- | --- | --- | --- | --- | --- | --- |
| **QOLIE-10** | 10-items  Epilepsy effects  (memory, physical effects, and mental effects of medication), Mental health (energy, depression, overall quality of life)  Role functioning (seizure worry, work, driving, social limits) | Patient | Good construct validity | Partial evidence (Wide range of Pearson correlation coefficient for different items (r = 0.48-0.81, all p< 0.001) suggesting some items may be less reliably upon re-testing | 4 weeks | (46,47) |
| **QOLIE-31** | 31-items  Emotional/Psychological Effects (seizure worry, overall QoL, emotional well-being, energy/fatigue subscales) and Medical/Social Effects (medication effects, work-driving-social limits, cognitive function subscales | Patient | Good internal consistency | Adequate responsiveness and test-retest reliability | 4 weeks | (47) |

1. **Behaviour**

Scores to measure behavioural disorders impacting daily life functioning and standardised measures of adaptive behaviour used as an indication of development can be considered as outcome measures in N-of-1 strategies for monogenic epilepsies. Two key limitations should be considered. First, for the majority of rare monogenic epilepsies we lack studies evaluating the properties of scores measured in that population. Therefore, the available scores may lack sensitivity or show floor or ceiling effects which limit interpretation of the results. This is exemplified in the study by Berg et al. 2021 showing limitations of the Vineland Adaptive Behaviour scale (VABS) for SCN2A-related developmental and epileptic encephalopathy (DEE) (48,49). Second, scores aiming to measure adaptive behaviour as a part of development often require an interval of at least 6 months between tests. Therefore, the VABS would not be suitable in an N-of-1 strategy. The Child Behavioural Checklist (CBCL) has been validated for a minimum interval of 6 months but has been used at shorter intervals (e.g. 12 weeks) (50,51). We recommend the following scores for clinical N-of-1 strategies.

**Table 8: Examples of behavioural scores suitable to score in short intervals**

| **Score** | **Population for which validated** | **User** | **Domains** | **Interval between tests, time to score** | **Reference** |
| --- | --- | --- | --- | --- | --- |
| Aberrant Behavioural Checklist  (ABC) | Validated for children and adults with intellectual disability and autism spectrum disorder | Proxy | Irritability  Lethargy  Stereotypic behaviour  Hyperactivity  Inappropriate speech | 4 weeks, 15 minutes | (48,52) |
| Strengths and Difficulties questionnaire (SDQ) | Age 2-17 (although variable age range use reported) | Proxy- and self-report (ages 11-16) | Emotional symptoms, conduct problems, hyperactivity/inattention, peer relationship problems, prosocial behaviour | 4 weeks, 15 minutes | (53–55) |
| Behaviour Assessment System for Children (BASC) | Age 2-21, validated for epilepsy | Proxy- and self- report | Externalizing problems, Internalizing problems, Adaptive skills | Unclear, BASC-Flex developed to monitor several times in long term | (56–58) |

**5. Speech/language**

Changes in speech and language are difficult to measure over short periods of time (<3 months). Most often these outcomes are neuropsychological tests, the regular use of which can be challenging in an N-of-1 strategy. Berg et al. 2022 summarised the psychometric characteristics of communication scores for children with developmental and epileptic encephalopathies (59). The Communication and Symbolic Behaviour Scales (CSBS) for infants under 18 months at an average re-test interval of 2 to 4 months could be suitable for N-of-1 strategies (59,60). The children communication checklist (CCC-2) may be suitable for children aged 4-16 years and could provide a pragmatic assessment of language impairment in children, rated by parent/caregiver, with a test re-test period of 28 days (61). However, this has not been validated in children with epilepsy.

**6. Motor function**

Commonly used scores for movement/motor disorders include the Scale for the Assessment and Rating of Ataxia (SARA) and the Global Motor Functional Measurement (GMFM-88). The SARA has been validated in patients with Friederich's ataxia, spinocerebellar ataxia, and early-onset ataxia (62–64). The interval used to assess test re-test reliability was 1 to 34 days (63). A recent crossover trial protocol uses an interval of 12 weeks between scores (65). A systematic review of available measurement tools in children with developmental central hypotonia has been published recently (66). Another systematic review addressed the properties of instruments for the evaluation of motor abilities for children with severe multiple disabilities, including GMFM-88 (67). To our knowledge no studies have validated the GMFM-88 specifically for individuals with DEEs.

**7. Cognition**

Cognition is a challenging outcome to measure repeatedly over short periods of time (<3 months) in N-of-1 strategies. However, executive function could be measured as a form of ‘fluid IQ.’ Many executive function tests are task-based and may be subject to learning bias if repeated several times at short intervals. For young children, the ages and stages questionnaire can be used to assess developmental progress in children up to the age of 5 ½ years. While this is not a direct measure of cognitive function and includes other domains such as social functioning and motor skills, it could be a useful score to screen and follow-up on development in young children. To our knowledge none of the scores below have been validated for children with DEEs or intellectual disability.

| **Score** | **Population for which validated** | **Reported by** | **Domains** | **Interval between tests, time to score** | **Reference** |
| --- | --- | --- | --- | --- | --- |
| EpiTrack | Children and teenagers with epilepsy 6 to 18 years old | Task-based, performed by patient, reported by neuropsychologist | Executive functioning:  Speed  Flexibility  Planning  Response inhibition  Word fluency  Working memory | 3 months, 15 minutes | (68,69) |
| Behaviour Rating Inventory of Executive Function (BRIEF)/BRIEF-Preschool version (BRIEF-P) | Children and adolescents with epilepsy ages 2-5 and 5-11 and 11-18 years old | Patient, parent, or teacher | Executive functioning:  Initiation  Inhibition  Shift  Emotional control  Monitor  Working memory  Plan/Organize | 6 months but in practice repeated in shorter intervals, 15 minutes | (70–73) |
| Ages and Stages questionnaire-3 (ASQ-3) | Infants and children from 1 to 66 months | Parent | Communication  Gross motor  Fine motor  Problem solving  Personal-social | Every 2 months, 15 minutes | (74) |

**8. Patient-specific outcomes**

The Goal Attainment Scale (GAS) is a patient-defined scale of most relevant symptoms, with points predefined based on patients’ goals in several domains: physical health and functioning, cognitive functioning, emotional and psychological well-being, social and interpersonal relationships, behaviour, daily living, and independence, occupational or academic performance, recreational and leisure activities, community involvement. Particularly for patients with rare disease or complex phenotypes, the GAS can offer a complementary alternative to generic outcome measurements (75).

The GAS has been applied regularly in clinical care by rehabilitation physicians (76) and showed complementary results when combined with standardised outcome measurements (77). Recommendations for appraisal of the use of GAS and training of healthcare professionals have been published (78,79).

**C. Statistical analysis**

The primary objective of N-of-1 strategies in clinical care is to assess the value of a treatment for an individual patient. The quality of collected data and effective communication of results are paramount to achieving this objective.

**9.1 Considerations for outcome analysis**

**9.1.1 Measurement frequency and effect size**

While simple non-parametric tests like the Wilcoxon signed-rank test can compare treatment effects, they fail to account for autocorrelation, time trends and repeated measurements within periods (1). More sophisticated statistical regression models are recommended to address these factors. Furthermore, traditional p-value from frequentist analyses may be less informative for clinical decision making than probabilistic estimates of achieving meaningful clinical effects. Bayesian inference aligns particularly well with clinical decision-making by providing probabilistic estimates based on prior beliefs updated with new evidence (80,81). For rare monogenic epilepsies, Bayesian modelling is strongly recommended. The relationship between outcome measurement frequency and expected effect size is crucial:

- Recurring events in N-of-1 strategies serve a similar function to sample size in conventional trial design;
- Paroxysmal, recurring high-frequency events (e.g., seizures) provide robust data for statistical analysis;
- Low frequency events require larger effect sizes to demonstrate statistical relevant effects (see section on seizure frequency and period duration);
- Single measurements per period (e.g., quality of life scores) provide less robust data due to limited temporal resolution.

**9.1.2 Outcome analysis methods**

Two key approaches for analysing outcomes include:

**Minimal clinically** **important difference (MCID):**

• Can be based on established literature standards (e.g., 50% seizure frequency reduction). However, despite the ubiquitous use of 50% seizure frequency reduction as a cut-off to indicate treatment responders in epilepsy trials, this generic cut-off has been widely criticised due to its doubtful relevance to QoL (82).

- It is recommended to individualise the MCID according to patient characteristics and wishes;
- It can be applied to posterior distributions in Bayesian analysis to compute probability of achieving MCID.

**Bayes Factor analysis:**

- Compares evidence for treatment effect (H1) against null hypothesis (H0);
- Expressed as likelihood ratio between models;
- Higher Bayes Factors indicate stronger evidence for treatment effect.

**9.1.3 Complementary nature of MCID and Bayes Factor**

While MCID and Bayes Factor analyses serve distinct purposes, they complement each other in providing a comprehensive evaluation of treatment effects. The Bayes Factor quantifies the statistical evidence for a treatment effect by comparing competing hypotheses and answering the question “Is there an effect?” However, it does not address the clinical relevance of this effect. Conversely, the MCID focuses on clinical significance by establishing whether the observed effect meets a predetermined threshold of meaningful change, thereby addressing the question “Is the effect large enough to matter?”

Using both approaches provides a more complete picture: the Bayes Factor can establish the presence of a treatment effect, while the MCID analysis determines whether this effect is clinically meaningful enough to warrant treatment implementation. For example, a treatment might show strong statistical evidence of effect (high Bayes Factor) but fail to meet the MCID threshold, suggesting that while the effect is real, it may not be clinically worthwhile.

**9. 2 Power analysis**

Power analysis in N-of-1 strategies focuses on repeated measurements rather than sample size. Simulation analyses can help in optimising the design for different patient characteristics, particularly in determining appropriate measurement frequencies and period durations (83).

**9. 3 Defining priors**

- The use of prior data is unique to Bayesian inference. The use of prior knowledge can influence the estimated treatment effects. Therefore, selection of Bayesian priors must be justified. Below a brief overview:
  - Non-informative: Results will be driven solely by the data (84,85);
  - Informative: Use of previously available data may influence the results in the direction of those data and therefore requires careful interpretation;
  - Weakly informative: Uses a balance between prior knowledge and new data.

| Example of methods to define informative Bayesian priors | | |
| --- | --- | --- |
| Method | Relevance | Reference |
| Based on data from randomized controlled trials (RCTs) (historical data) | - Results from prior RCTs can be used to elicit priors. - The historical control group data selected as prior must be comparable to the data being analysed: patient characteristics, reference treatment and effectiveness of reference treatment. | (85–88) |
| Based on expert opinion | - Interviews with clinicians, questionnaires and focus groups with experts can be used. - A team of experts can help estimate the expected treatment effect size for a patient with specific characteristics. - As a drawback, experts may be subject to bias | (89) |
| Combination of the above | - Combining results of RCTs and expert opinion provides a consistency check between priors elicited with both methods. | (85,90) |

**9.4 Statistical software to analyse results**

Several statistical packages are available for analysing data from N-of-1 strategies:

1. R Packages:

- brms (Bayesian Regression Models using Stan)

1. Specialized Software:

- JASP (user-friendly Bayesian analysis)
- Stan (flexible Bayesian modelling)
- WinBUGS/OpenBUGS (Bayesian inference)

For implementation, we recommend using R with the brms package for Bayesian analyses due to its flexibility and robust modelling capabilities. JASP provides a more accessible option for researchers less familiar with programming. Complex designs may require custom implementation in Stan or similar frameworks.

The choice of software should be guided by the specific requirements of the trial design, the complexity of the analysis, and the expertise of the research team.

**References**

1. DEcIDE Methods Center N-of-1 Guidance Panel (Duan N, Eslick I, Gabler NB, Kaplan HC, Kravitz RL, Larson EB, Pace WD, Schmid CH, Sim I VS. Design and Implementation of N-of- 1 Trials: A User’s Guide [Internet]. No. 13(14). Kravitz R, Duan N, editors. Rockville, MD: Agency for Healthcare Research and Quality; 2014. Available from: www.effectivehealthcare.ahrq.gov/N-1-Trials.cfm

2. Margolis A, Giuliano C. Making the switch: From case studies to N-of-1 trials. Epilepsy Behav Rep [Internet]. 2019;12:100336. Available from: https://doi.org/10.1016/j.ebr.2019.100336

3. Guyatt G, Sackett D, Adachi J, Chong J, Rosenbloom D, Pharm D. A clinician’s guide for conducting randomized trials in individual patients. CMAJ. 1988;(139):497–503.

4. Seiden LG, Connor GS. The importance of drug titration in the management of patients with epilepsy. Epilepsy and Behavior. 2022 Mar 1;128:e108517.

5. Defelippe VM, Brilstra EH, Otte WM, Cross HJ, O’Callaghan F, De Giorgis V, et al. N-of-1 trials in epilepsy: A systematic review and lessons paving the way forward. Epilepsia. John Wiley and Sons Inc; 2024.

6. Brandon Westover M, Cormier J, Bianchi MT, Shafi M, Kilbride R, Cole AJ, et al. Revising the “rule of Three” for inferring seizure freedom. Epilepsia. 2012 Feb;53(2):368–76.

7. Friedrichs-Maeder C, Proix T, Tcheng TK, Skarpaas T, Rao VR, Baud MO. Seizure Cycles under Pharmacotherapy. Ann Neurol. 2024 Apr 1;95(4):743–53.

8. Karoly PJ, Rao VR, Gregg NM, Worrell GA, Bernard C, Cook MJ, et al. Cycles in epilepsy. Vol. 17, Nature Reviews Neurology. Nature Research; 2021. p. 267–84.

9. Goldenholz DM, Goldenholz EB, Kaptchuk TJ. Quantifying and controlling the impact of regression to the mean on randomized controlled trials in epilepsy. Epilepsia. 2023 Oct 1;64(10):2635–43.

10. Goldenholz DM, Strashny A, Cook M, Moss R, Theodore WH. A multi-dataset time-reversal approach to clinical trial placebo response and the relationship to natural variability in epilepsy. Seizure. 2017 Dec 1;53:31–6.

11. Schulze-Bonhage A, Richardson MP, Brandt A, Zabler N, Dümpelmann M, San Antonio-Arce V. Cyclical underreporting of seizures in patient-based seizure documentation. Ann Clin Transl Neurol. 2023 Oct 1;10(10):1863–72.

12. Zabler N, Swinnen L, Biondi A, Novitskaya Y, Schütz E, Epitashvili N, et al. High precision in epileptic seizure self-reporting with an app diary. Sci Rep. 2024 Dec 1;14(1).

13. Fisher RS, Blum DE, DiVentura B, Vannest J, Hixson JD, Moss R, et al. Seizure diaries for clinical research and practice: Limitations and future prospects. Vol. 24, Epilepsy and Behavior. Academic Press Inc.; 2012. p. 304–10.

14. Buchhalter J, Neuray C, Cheng JY, D’Cruz ON, Datta AN, Dlugos D, et al. EEG parameters as endpoints in epilepsy clinical trials - An expert panel opinion paper. Epilepsy Res. 2022 Nov 1;187.

15. Auvin S, Nortvedt C, Fuller DS, Sahebkar F. Seizure-free days as a novel outcome in patients with Lennox–Gastaut syndrome: Post hoc analysis of patients receiving cannabidiol in two randomized controlled trials. Epilepsia. 2023 Jul 1;64(7):1812–20.

16. Mytinger JR, Hussain SA, Islam MP, Millichap JJ, Patel AD, Ryan NR, et al. Improving the inter-rater agreement of hypsarrhythmia using a simplified EEG grading scale for children with infantile spasms. Epilepsy Res. 2015 Oct 1;116:93–8.

17. Hussain SA, Kwong G, Millichap JJ, Mytinger JR, Ryan N, Matsumoto JH, et al. Hypsarrhythmia assessment exhibits poor interrater reliability: A threat to clinical trial validity. Epilepsia. 2015 Jan 1;56(1):77–81.

18. Deckard E, Sathe R, Tabibzadeh D, Terango A, Groves A, Rajaraman RR, et al. Epileptic spasms relapse is associated with response latency but not conventional attributes of post-treatment EEG. Epilepsia Open. 2024 Jun 1;9(3):1034–41.

19. Yuskaitis CJ, Mytinger JR, Baumer FM, Zhang B, Liu S, Samanta D, et al. Association of Time to Clinical Remission with Sustained Resolution in Children with New-Onset Infantile Spasms. Neurology. 2022 Nov 29;99(22):E2494–503.

20. Mytinger JR, Vidaurre J, Moore-Clingenpeel M, Stanek JR, Albert DVF. A reliable interictal EEG grading scale for children with infantile spasms – The 2021 BASED score. Epilepsy Res. 2021 Jul 1;173.

21. Van Den Munckhof B, Van Dee V, Sagi L, Caraballo RH, Veggiotti P, Liukkonen E, et al. Treatment of electrical status epilepticus in sleep: A pooled analysis of 575 cases. Epilepsia. 2015 Nov 1;56(11):1738–46.

22. Buzatu M, Bulteau C, Altuzarra C, Dulac O, Van Bogaert P. Corticosteroids as treatment of epileptic syndromes with continuous spike-waves during slow-wave sleep. In: Epilepsia. 2009. p. 68–72.

23. van Arnhem MML, van den Munckhof B, Arzimanoglou A, Perucca E, Metsähonkala L, Rubboli G, et al. Corticosteroids versus clobazam for treatment of children with epileptic encephalopathy with spike-wave activation in sleep (RESCUE ESES): a multicentre randomised controlled trial. Lancet Neurol. 2024 Feb 1;23(2):147–56.

24. Beniczky S, Aurlien H, Brøgger JC, Hirsch LJ, Schomer DL, Trinka E, et al. Standardized computer-based organized reporting of EEG: SCORE – Second version. Vol. 128, Clinical Neurophysiology. Elsevier Ireland Ltd; 2017. p. 2334–46.

25. Reus EEM, Visser GH, Cox FME. Determining the Spike-Wave Index Using Automated Detection Software. Journal of Clinical Neurophysiology. 2021 May 1;38(3):198–201.

26. Joshi CN, Chapman KE, Bear JJ, Wilson SB, Walleigh DJ, Scheuer ML. Semiautomated spike detection software persyst 13 is noninferior to human readers when calculating the spike-wave index in electrical status epilepticus in sleep. Journal of Clinical Neurophysiology. 2018;35(5):370–4.

27. Birbeck GL, Hays RD, Cui X, Vickrey BG. Seizure reduction and quality of life improvements in people with epilepsy. Epilepsia. 2002;43(5):535–8.

28. Luoni C, Bisulli F, Canevini MP, De Sarro G, Fattore C, Galimberti CA, et al. Determinants of health-related quality of life in pharmacoresistant epilepsy: Results from a large multicenter study of consecutively enrolled patients using validated quantitative assessments. Epilepsia. 2011 Dec;52(12):2181–91.

29. Ferro MA. Risk factors for health-related quality of life in children with epilepsy: A meta-analysis. Epilepsia. 2014 Nov 1;55(11):1722–31.

30. Hulse D, Harvey AS, Freeman JL, Mackay MT, Dabscheck G, Barton SM. Clinical application of the PedsQL Epilepsy Module (PedsQL-EM) in an ambulatory pediatric epilepsy setting. Epilepsy and Behavior. 2020 May 1;106.

31. Mitchell JW, Sossi F, Miller I, Jaber PB, Das-Gupta Z, Fialho LS, et al. Development of an International Standard Set of Outcomes and Measurement Methods for Routine Practice for Adults with Epilepsy: The International Consortium for Health Outcomes Measurement Consensus Recommendations. Epilepsia. 2024 Jul 1;65(7):1916–37.

32. Mitchell JW, Sossi F, Miller I, Jaber PB, Das-Gupta Z, Fialho LS, et al. Development of an International Standard Set of Outcomes and Measurement Methods for Routine Practice for Infants, Children, and Adolescents with Epilepsy: The International Consortium for Health Outcomes Measurement Consensus Recommendations. Epilepsia. 2024 Jul 1;65(7):1938–61.

33. Sarlo GL, Haughton T, Rizakos E, Merwin S, Havens KA, Pasupuleti A, et al. Comparison of psychosocial screeners in an epilepsy clinic. Epilepsy and Behavior. 2023 Nov 1;148.

34. Modi AC, Junger KF, Mara CA, Kellermann T, Barrett L, Wagner J, et al. Validation of the PedsQL Epilepsy Module: A pediatric epilepsy-specific health-related quality of life measure. Epilepsia. 2017 Nov 1;58(11):1920–30.

35. Borghs S, de la Loge C, Cramer JA. Defining minimally important change in QOLIE-31 scores: Estimates from three placebo-controlled lacosamide trials in patients with partial-onset seizures. Epilepsy and Behavior. 2012 Mar;23(3):230–4.

36. Wiebe S, Eliasziw M, Matijevic S. Changes in quality of life in epilepsy: How large must they be to be real? Epilepsia. 2001;42(1):113–8.

37. Wiebe S, Matijevic S, Eliasziw M. Clinically important change in quality of life in epilepsy [Internet]. Vol. 73, J Neurol Neurosurg Psychiatry. 2002. Available from: www.jnnp.com

38. Crudgington H, Rogers M, Morris H, Gringras P, Pal DK, Morris C. Epilepsy-specific patient-reported outcome measures of children’s health-related quality of life: A systematic review of measurement properties. Epilepsia. 2020;61(2):230–48.

39. Breau GM, Camfield CS, Camfield PR, Breau LM. Evaluation of the responsiveness of the Impact of Pediatric Epilepsy Scale. Epilepsy and Behavior. 2008 Oct;13(3):454–7.

40. Camfield C, Breau L, Camfield P. Impact of pediatric epilepsy on the family: A new scale for clinical and research use. Epilepsia. 2001;42(1):104–12.

41. Follansbee-Junger KW, Mann KA, Guilfoyle SM, Morita DA, Varni JW, Modi AC. Development of the PedsQL^TM^ Epilepsy Module: Focus group and cognitive interviews. Epilepsy and Behavior. 2016 Sep 1;62:115–20.

42. Goodwin SW, Ferro MA, Speechley KN. Development and assessment of the Quality of Life in Childhood Epilepsy Questionnaire (QOLCE-16). Epilepsia. 2018 Mar 1;59(3):668–78.

43. Puka K, Goodwin SW, Ferro MA, Smith M Lou, Widjaja E, Anderson KK, et al. Validation of the Quality of Life in Childhood Epilepsy Questionnaire (QOLCE-55 and QOLCE-16) for use by parents of young adults with childhood-onset epilepsy. Epilepsy and Behavior. 2020 Mar 1;104.

44. Ronen GM, Streiner DL, Rosenbaum P. Health-related quality of life in children with epilepsy: development and validation of self-report and parent proxy measures. Epilepsia. 2003;44(4):598–612.

45. Verhey LH, Kulik DM, Ronen GM, Rosenbaum P, Lach L, Streiner DL. Quality of life in childhood epilepsy: What is the level of agreement between youth and their parents? Epilepsy and Behavior. 2009 Feb;14(2):407–10.

46. Cramer JA, Perrine K, Devinsky O, Meador K. A brief questionnaire to screen for quality of life in epilepsy: The QOLIE-10. Epilepsia. 1996 Jun;37(6):577–82.

47. Cramer JA, Arrigo C, Ve G, Hammée V, Bromfield EB, Cramer JA. Comparison between the QOLIE-31 and derived QOLIE-10 in a clinical trial of levetiracetam [Internet]. Vol. 41, Epilepsy Research. 2000. Available from: www.elsevier.com/locate/epilepsyres

48. Kaat AJ, Zelko F, Wilkening G, Berg AT. Evaluation of the Aberrant Behavior Checklist for Developmental and Epileptic Encephalopathies. Epilepsy and Behavior. 2021 Jun 1;119.

49. Berg AT, Smith SN, Frobish D, Beckerman B, Levy SR, Testa FM, et al. Longitudinal assessment of adaptive behavior in infants and young children with newly diagnosed epilepsy: Influences of etiology, syndrome, and seizure control. Pediatrics. 2004;114(3):645–50.

50. Paolicchi JM, Ross G, Lee D, Drummond R, Isojarvi J. Clobazam and Aggression-Related Adverse Events in Pediatric Patients with Lennox-Gastaut Syndrome. Pediatr Neurol. 2015 Oct 1;53(4):338–42.

51. de la Loge C, Hunter SJ, Schiemann J, Yang H. Assessment of behavioral and emotional functioning using standardized instruments in children and adolescents with partial-onset seizures treated with adjunctive levetiracetam in a randomized, placebo-controlled trial. Epilepsy and Behavior. 2010 Jul;18(3):291–8.

52. Rojahn J, Schroeder SR, Mayo-Ortega L, Oyama-Ganiko R, LeBlanc J, Marquis J, et al. Validity and reliability of the Behavior Problems Inventory, the Aberrant Behavior Checklist, and the Repetitive Behavior Scale - Revised among infants and toddlers at risk for intellectual or developmental disabilities: A multi-method assessment approach. Res Dev Disabil. 2013 May;34(5):1804–14.

53. Goodman R. The strengths and difficulties questionnaire: A research note. J Child Psychol Psychiatry. 1997 Jul;38(5):581–6.

54. Kaiser S, Halvorsen MB. The Strengths and Difficulties Questionnaire self-report-, parent-, and teacher version in children with intellectual and developmental disabilities. Res Dev Disabil. 2022 Apr 1;123.

55. Goodman R, Scott S. Comparing the Strengths and Difficulties Questionnaire and the Child Behavior Checklist: Is Small Beautiful? Vol. 27, Journal of Abnormal Child Psychology. 1999.

56. Allison Bender H, Auciello D, Morrison CE, MacAllister WS, Zaroff CM. Comparing the convergent validity and clinical utility of the Behavior Assessment System for Children-Parent Rating Scales and Child Behavior Checklist in children with epilepsy. Epilepsy and Behavior. 2008 Jul;13(1):237–42.

57. Altmann RA, Reynolds CR, Kamphaus RW, Vannest KJ. BASC-3. In: Encyclopedia of Clinical Neuropsychology. Springer International Publishing; 2018. p. 1–7.

58. Clary LE, Vander Wal JS, Titus JB. Examining health-related quality of life, adaptive skills, and psychological functioning in children and adolescents with epilepsy presenting for a neuropsychological evaluation. Epilepsy and Behavior. 2010 Nov;19(3):487–93.

59. Berg AT, Kaat AJ, Zelko F, Wilkening G. Rare diseases – rare outcomes: Assessing communication abilities for the developmental and epileptic encephalopathies. Epilepsy and Behavior. 2022 Mar 1;128.

60. Wetherby AM, Allen L, Cleary J, Kublin K, Goldstein H. Validity and Reliability of the Communication and Symbolic Behavior Scales Developmental Profile With Very Young Children. Journal of Speech, Language, and Hearing Research [Internet]. 2002 Dec;45(6):1202–18. Available from: http://pubs.asha.org/doi/10.1044/1092-4388%282002/097%29

61. Bishop DVM, Baird G. Parent and teacher report of pragmatic aspects of communication: Use of the Children’s Communication Checklist in a clinical setting. Dev Med Child Neurol. 2001;43(12):809–18.

62. Lawerman TF, Brandsma R, Maurits NM, Martinez-Manzanera O, Verschuuren-Bemelmans CC, Lunsing RJ, et al. Paediatric motor phenotypes in early-onset ataxia, developmental coordination disorder, and central hypotonia. Dev Med Child Neurol. 2020 Jan 1;62(1):75–82.

63. Schmitz-Hübsch T, du Montcel ST, Baliko L, Boesch S, Bonato S, Fancellu R, et al. Reliability and validity of the International Cooperative Ataxia Rating Scale: A study in 156 spinocerebellar ataxia patients. Movement Disorders. 2006;21(5):699–704.

64. Bürk K, Mälzig U, Wolf S, Heck S, Dimitriadis K, Schmitz-Hübsch T, et al. Comparison of three clinical rating scales in Friedreich Ataxia (FRDA). Movement Disorders. 2009 Sep 15;24(12):1779–84.

65. Fields T, M. Bremova T, Billington I, Churchill G, Evans W, Fields C, et al. N-acetyl-L-leucine for Niemann-Pick type C: a multinational double-blind randomized placebo-controlled crossover study. Trials. 2023 Dec 1;24(1).

66. Hidalgo Robles Á, Paleg GS, Livingstone RW. Identifying and Evaluating Young Children with Developmental Central Hypotonia: An Overview of Systematic Reviews and Tools. Vol. 12, Healthcare (Switzerland). Multidisciplinary Digital Publishing Institute (MDPI); 2024.

67. Mensch SM, Rameckers EAA, Echteld MA, Evenhuis HM. Instruments for the evaluation of motor abilities for children with severe multiple disabilities: A systematic review of the literature. Vol. 47, Research in Developmental Disabilities. Elsevier Inc.; 2015. p. 185–98.

68. Lähde N, Basnyat P, Lehtinen H, Rainesalo S, Rosti-Otajärvi E, Peltola J. EpiTrack is a feasible tool for assessing attention and executive functions in patients with refractory epilepsy. Epilepsy and Behavior. 2021 Feb 1;115.

69. Helmstaedter C, Schoof K, Rossmann T, Reuner G, Karlmeier A, Kurlemann G. Introduction and first validation of EpiTrack Junior, a screening tool for the assessment of cognitive side effects of antiepileptic medication on attention and executive functions in children and adolescents with epilepsy. Epilepsy and Behavior. 2010 Sep;19(1):55–64.

70. Maiman M, Salinas CM, Gindlesperger MF, Westerveld M, Vasserman M, MacAllister WS. Utility of the Behavior Rating Inventory of Executive Function–Preschool version (BRIEF-P) in young children with epilepsy. Child Neuropsychology. 2018 Oct 3;24(7):975–85.

71. Hauger LE, Lossius MI, Aaberg KM, Helmstaedter C, Lossius J, Skogan AH. Screening of attention and executive functions in pediatric patients at a tertiary epilepsy center. European Journal of Paediatric Neurology. 2023 Sep 1;46:35–41.

72. Slick D, Lautzenhiser A, Sherman E, Eyrl K. Frequency of scale elevations and factor structure of the Behavior Rating Inventory of Executive Function (BRIEF) in children and adolescents with intractable epilepsy. Child Neuropsychology. 2006 Jul 1;12(3):181–9.

73. Gioia GA, Isquith PK, Guy SC, Kenworthy L, Baron IS. Behavior rating inventory of executive function. Vol. 6, Child Neuropsychology. Swets en Zeitlinger B.V.; 2000. p. 235–8.

74. Squires J, Bricker D, Potter L. Revision of a Parent-Completed Developmental Screening Tool: Ages and Stages Questionnaires 1 [Internet]. Vol. 22, Journal of Pediatric Psychology. 1997. Available from: https://academic.oup.com/jpepsy/article/22/3/313/917423

75. Gaasterland CMW, Van Der Weide MCJ, Roes KCB, Van Der Lee JH. Goal attainment scaling as an outcome measure in rare disease trials: A conceptual proposal for validation. BMC Med Res Methodol. 2019;19(1):1–10.

76. Harpster K, Sheehan A, Foster EA, Leffler E, Schwab SM, Angeli JM. The methodological application of goal attainment scaling in pediatric rehabilitation research: a systematic review. Disabil Rehabil. 2019 Nov 20;41(24):2855–64.

77. Steenbeek D, Gorter JW, Ketelaar M, Galama K, Lindeman E. Responsiveness of goal attainment scaling in comparison to two standardized measures in outcome evaluation of children with cerebral palsy. Clin Rehabil. 2011 Dec;25(12):1128–39.

78. Krasny-Pacini A, Evans J, Sohlberg MM, Chevignard M. Proposed criteria for appraising goal attainment scales used as outcome measures in rehabilitation research. Vol. 97, Archives of Physical Medicine and Rehabilitation. W.B. Saunders; 2016. p. 157–70.

79. Logan B, Viecelli AK, Pascoe EM, Pimm B, Hickey LE, Johnson DW, et al. Training healthcare professionals to administer Goal Attainment Scaling as an outcome measure. J Patient Rep Outcomes. 2024 Dec 1;8(1).

80. Ursino M, Stallard N. Bayesian approaches for confirmatory trials in rare diseases: Opportunities and challenges. Int J Environ Res Public Health. 2021;18(3):1–9.

81. Kidwell KM, Roychoudhury S, Wendelberger B, Scott J, Moroz T, Yin S, et al. Application of Bayesian methods to accelerate rare disease drug development: scopes and hurdles. Vol. 17, Orphanet Journal of Rare Diseases. BioMed Central Ltd; 2022.

82. Perucca E, Wiebe S. Not all that glitters is gold: A guide to the critical interpretation of drug trials in epilepsy. Epilepsia Open. 2016;1(1–2):9–21.

83. KS O, JB C, Fahey M, JL F, IE S, Gillam L, et al. Protocol for a single patient therapy plan: A randomised, double-blind, placebo-controlled N-of-1 trial to assess the efficacy of cannabidiol in patients with intractable epilepsy. J Paediatr Child Health [Internet]. 2020;56(12):1918–23. Available from: https://pubmed.ncbi.nlm.nih.gov/32965057/

84. Zucker DR, Schmid CH, McIntosh MW, D’Agostino RB, Selker HP, Lau J. Combining single patient (N-of-1) trials to estimate population treatment effects and to evaluate individual patient responses to treatment. J Clin Epidemiol. 1997;50(4):401–10.

85. Stunnenberg BC, Woertman W, Raaphorst J, Statland JM, Griggs RC, Timmermans J, et al. Combined N-of-1 trials to investigate mexiletine in non-dystrophic myotonia using a Bayesian approach; study rationale and protocol. BMC Neurol. 2015;15(1):1–10.

86. Eggleston BS, Ibrahim JG, McNeil B, Catellier D. BayesCTDesign: An R Package for Bayesian Trial Design Using Historical Control Data. J Stat Softw. 2021;100.

87. Psioda MA, Ibrahim JG. Bayesian clinical trial design using historical data that inform the treatment effect. Biostatistics. 2019 Jul 1;20(3):400–15.

88. Zucker DR, Ruthazer R, Schmid CH, Feuer JM, Fischer PA, Kieval RI, et al. Lessons learned combining N-of-1 trials to assess fibromyalgia therapies. Journal of Rheumatology. 2006;33(10):2069–77.

89. Spiegelhalter DJ, Abrams KR, Myles JP. Prior Distributions. In: Bayesian Approaches to Clinical Trials and Health‐Care Evaluation. Wiley; 2003. p. 139–80.

90. Stunnenberg BC, Raaphorst J, Groenewoud HM, Statland JM, Griggs RC, Woertman W, et al. Effect of Mexiletine on Muscle Stiffness in Patients with Nondystrophic Myotonia Evaluated Using Aggregated N-of-1 Trials. JAMA - Journal of the American Medical Association. 2018;320(22):2344–53.
